# Culture-expanded mesenchymal stromal cell therapy: does it work in knee osteoarthritis? A pathway to clinical success

**DOI:** 10.1038/s41423-023-01020-1

**Published:** 2023-04-25

**Authors:** Griffin Copp, Kevin P. Robb, Sowmya Viswanathan

**Affiliations:** 1 Osteoarthritis Research Program, Division of Orthopedic Surgery, Schroeder Arthritis Institute, University Health Network, Toronto, ON Canada; 2 Krembil Research Institute, University Health Network, Toronto, ON Canada; 3 Institute of Biomedical Engineering, University of Toronto, Toronto, ON Canada; 4 Department of Medicine, Division of Hematology, University of Toronto, Toronto, ON Canada

**Keywords:** MSC, Osteoarthritis, Cell therapy, mesenchymal stromal cell, RCT, Immunology, Inflammation

## Abstract

Osteoarthritis (OA) is a degenerative multifactorial disease with concomitant structural, inflammatory, and metabolic changes that fluctuate in a temporal and patient-specific manner. This complexity has contributed to refractory responses to various treatments. MSCs have shown promise as multimodal therapeutics in mitigating OA symptoms and disease progression. Here, we evaluated 15 randomized controlled clinical trials (RCTs) and 11 nonrandomized RCTs using culture-expanded MSCs in the treatment of knee OA, and we found net positive effects of MSCs on mitigating pain and symptoms (improving function in 12/15 RCTs relative to baseline and in 11/15 RCTs relative to control groups at study endpoints) and on cartilage protection and/or repair (18/21 clinical studies). We examined MSC dose, tissue of origin, and autologous vs. allogeneic origins as well as patient clinical phenotype, endotype, age, sex and level of OA severity as key parameters in parsing MSC clinical effectiveness. The relatively small sample size of 610 patients limited the drawing of definitive conclusions. Nonetheless, we noted trends toward moderate to higher doses of MSCs in select OA patient clinical phenotypes mitigating pain and leading to structural improvements or cartilage preservation. Evidence from preclinical studies is supportive of MSC anti-inflammatory and immunomodulatory effects, but additional investigations on immunomodulatory, chondroprotective and other clinical mechanisms of action are needed. We hypothesize that MSC basal immunomodulatory “fitness” correlates with OA treatment efficacy, but this hypothesis needs to be validated in future studies. We conclude with a roadmap articulating the need to match an OA patient subset defined by molecular endotype and clinical phenotype with basally immunomodulatory “fit” or engineered-to-be-fit-for-OA MSCs in well-designed, data-intensive clinical trials to advance the field.

## Introduction

### Overview of osteoarthritis

Osteoarthritis (OA) is the most common form of arthritis, affecting an estimated 650 million people (654.1 [95% CI, 565.6–745.6]) aged 40 and older worldwide as of 2020, with an incidence of 203 per 10,000 person-years [95% CI, 206–331] [1]. The global age-standardized prevalence of knee OA is estimated to be 3.8%, with a higher prevalence in females than in males [2]. Globally, among the total OA cases in 2019, the knee was the leading anatomic site, accounting for 60.6% of all OA cases [3], and knee OA will be the focus of this review.

OA imposes socioeconomic burdens due to its morbidity, which results in reduced daily activity and productivity. Currently, arthritis costs an estimated US$ 24 billion annually in Canada, with projected increases to US$ 49 billion annually by 2031 [4], and US$ 140 billion annually in the United States of America [5]. Patients with OA experience a significant reduction in their quality of life, yet there are no disease-modifying treatments for this disease. Current treatments include lifestyle changes (exercise, weight loss, and quitting smoking); symptom-modifying injectables [6], including corticosteroids, hyaluronic acid (HA), platelet-rich plasma (PRP); and recently, autologous patient serum containing activated monocyte/macrophages (a mixed population termed as MΦs) [7] that are injected with short- to medium-term analgesic effects. OA is managed with these symptom-modifying approaches until surgical joint replacement can be performed—which is reserved for patients with end-stage OA—leaving a typical 10–20-year gap with limited clinical management of OA morbidity. Joint replacement for OA has a large effect size but is appropriate only at advanced disease stages [8]. With the paucity of effective treatments, there is an urgent need for clinical studies of new and existing therapies.

OA is a multifactorial disease affecting articular cartilage, subchondral bone, menisci (in the knee), synovial tissue (a membrane lining the joint and heterogeneously composed of fibroblasts, MΦs, and endothelial and stromal cells), tendon/ligaments and muscle [9]. While historically OA has been considered a disease of mechanical “wear-and-tear” that leads to the degradation of articular cartilage, more recently, this view has shifted to recognize the important roles of inflammation, metabolic dysregulation, and fibrosis in the complex disease pathophysiology and symptomology. Notably, these pathophysiological mechanisms are interrelated, and while in this review we emphasize the importance of modulating inflammation, we also include a discussion of other disease factors due to this complex interplay.

OA is highly heterogeneous in terms of clinical features and responses to available treatments, as well as through the contribution of different biochemical factors that contribute to the progressive and gradual loss of articular cartilage [9, 10] and bone changes and inflammation that cause joint pain and swelling, which impair patient mobility and quality of life [11]. As such, OA is now better understood not as a single disease but as a collection of different clinical phenotypes [12] with different and/or overlapping molecular endotypes that drive the disease; patients thus present with different combinations of pain, symptoms and dysfunction [13]. Mechanical instability [14, 15], trauma [16], sex [17, 18], age [19–21], obesity [22, 23] and metabolic syndrome [24–26] are major risk factors for knee OA. Within articular cartilage, biomechanical and chemical cues prompt a shift toward a proinflammatory and catabolic (“activated”) chondrocyte phenotype associated with chondrocyte hypertrophy and apoptosis that contribute to cartilage degradation [27–29]. The subchondral bone in OA is characterized by microcracks, bone marrow lesions, neovascularization, and osteophyte formation; this facilitates crosstalk between osteocytes and chondrocytes at the bone-cartilage interface, which is thought to play an important role in regulating the activated chondrocyte phenotype [30–32]. Synovial tissue, consisting primarily of synovial fibroblasts and MΦs [33, 34], is a key source of proinflammatory, catabolic, and profibrotic factors [35–37]. These factors contribute to inflammation, cartilage degradation, and fibrocartilage formation in articular cartilage and joint menisci, leading to joint swelling, stiffness, and pain [35–37]. The synovium becomes increasingly vascularized in OA, allowing increased immune cell infiltration into the joint that further propagates the inflammatory and degradative process [38, 39].

Inflammation plays a critical role in OA, and proinflammatory cytokines such as interleukin-1-beta (IL-1β), IL-6 and tumor necrosis factor alpha (TNF-α) are implicated in OA pathogenesis [40] and are elevated in OA joint tissues and synovial fluid [41, 42]. The joint-specific presence of these proinflammatory mediators drives cartilage catabolism and degradation, resulting in the recruitment of more immune cell infiltrates and perpetuating a negative cascade of increasing inflammation and patient pain and symptoms [43]. Targeting inflammatory mediators in OA is thus key to modifying disease progression [13]. Nonetheless, several single anti-inflammatory therapies, including anti-TNF [10] and anti-IL-1 [11, 12], have failed in clinical trials, although both TNF-α and IL-1β indisputably play key roles in OA pathogenesis. Tellingly, in a systematic review to classify patients based on clinical phenotypes, only 12% of patients fell into the inflammatory phenotype category [12], but this is likely reflective of the temporal nature of OA rather than the limited importance of proinflammatory cytokines in driving molecular endotypes [42]. The temporal, overlapping and heterogeneous complexity of this multi-tissue disease emphasizes the challenges of targeting single pathways for overall treatment effects. Multimodal disease-mitigating agents or combinations of agents are needed and should be matched appropriately to the temporal stage and patient endotype, representing significant headwinds in developing effective OA therapeutics.

### Mesenchymal stromal cells (MSCs) for the treatment of OA

MSCs are attractive therapeutic candidates for OA due to their multimodal mechanisms of action, including their immunomodulatory properties. MSCs modulate the inflammatory cytokine milieu and immune cell responses in the joint via the release of secreted paracrine factors and extracellular vesicles (EVs) and through host macrophage-mediated efferocytosis [44–47]. In addition to their immunomodulatory functions, MSCs exert regenerative effects through the release of growth factors, cytokines, organelle transfer, and other molecules that can mediate endogenous repair responses in the host microenvironment. MSCs derived from multiple sources, such as bone marrow, adipose tissue and the umbilical cord, have demonstrated unambiguous preclinical therapeutic efficacy in a wide variety of disease indications on account of these immunomodulatory and reparative properties. MSCs have been investigated in over 5000 patients with a demonstrable safety profile [46–48]. Currently, 10 MSC-based therapies, including Stempeucel®, CARTISTEM®, Prochymal®, Stemirac®, Alofisel®, and others, are approved in certain countries, and more than 1050 clinical trials are registered at clinicaltrials.gov for MSCs targeting multiple disease indications [49–51]. Notably, this includes CARTISTEM®, an allogeneic umbilical cord blood (CB) MSC product by MEDIPOST, approved in S. Korea for the treatment of degenerative arthritis and cartilage defects [52]. Importantly, there are no approved MSC products in the United States of America [53]. Thus, despite the existence of examples of successful global commercial MSC products, including the approval of StemOne®, an allogeneic cell therapy treatment for knee OA approved by the Drug Controller General of India in September 2022 [54], there is also evidence of mixed clinical effectiveness, which has limited commercial success in major markets. With respect to OA, MSC treatment, as assessed in several randomized clinical trials and meta-analyses, improves pain, function, quality of life scores and cartilage volume [46–50, 55], but data are based on limited sample sizes, and larger, high-powered trials are needed to reach definitive conclusions.

In this review, we will examine a curated set of peer-reviewed RCTs and discuss clinical evidence of efficacy from these controlled trials, focusing only on culture-expanded MSCs in treating OA [56–71]. Next, we will deconstruct the mechanisms of action of MSCs in OA and the role of MSC basal immunomodulatory fitness on therapeutic efficacy, highlighting the immunomodulatory role of MSCs and interplay with cartilage-reparative, antifibrotic and angiogenic effects. There are several reviews discussing preclinical MSC mechanisms of action in the treatment of OA [72–75], and we therefore focus our analysis primarily on clinical evidence. We will conclude with a roadmap that incorporates a discussion on stratifying inflammatory OA patients on the basis of baseline clinical phenotypes and disease endotypes, selecting and designing fit and fit-for-purpose MSCs, and incorporating appropriate clinical trial designs as critical next steps in advancing successful MSC clinical trials and enabling commercial MSC therapeutics for knee OA.

## Culture-expanded MSCs and randomized, controlled OA clinical trial results

### Selection criteria for the inclusion of randomized controlled trials (RCTs)

**Fig. 1:**
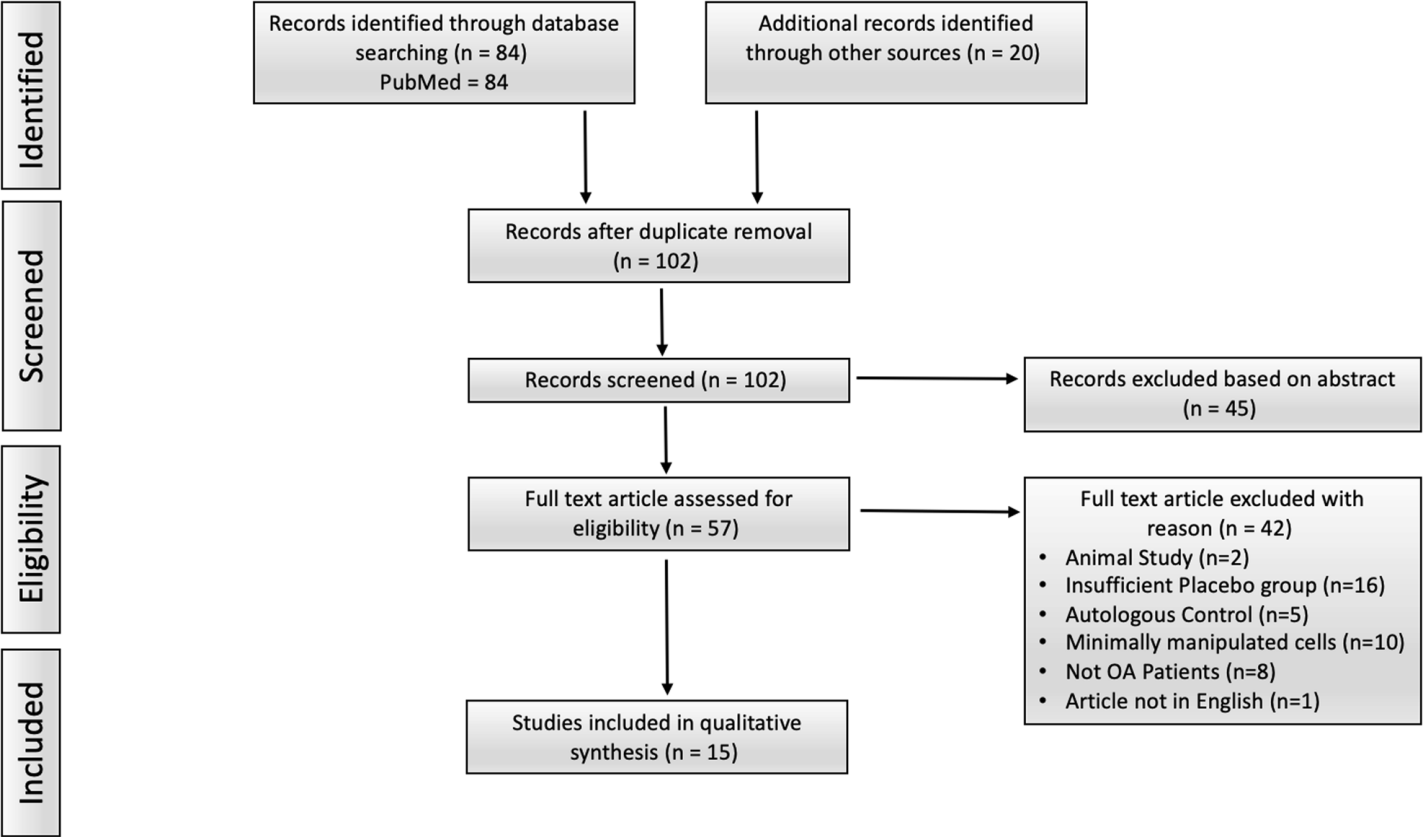
PRISMA flow diagram of the literature selection process. PubMed search string “((((((MPC) OR (Mesenchymal stem cell)) OR (MSC)) OR (ASC)) OR (Mesenchymal stromal cell)) AND (Osteoarthritis) + Clinical Trial + RCT”

To stringently probe the clinical efficacy of MSC therapies from a larger collection of reported data, we collected published data on RCTs for MSC effects in OA. We reviewed clinical trials from the PubMed database using the following search string: “mesenchymal stromal cell” or “mesenchymal stem cell” or “MSC” or “ASC” (adipose stromal/stem cells) or “MPC” (multipotent precursor/progenitor cell) and “osteoarthritis”. The results were further filtered by “Clinical Trial” and “Randomized Controlled Trial”, resulting in 84 clinical trials. Additionally, recently published meta-analyses [76–80] were cross-referenced to ensure the inclusion of all relevant clinical studies, bringing the total to 102 clinical trials once duplicates were removed. Next, abstracts were screened, and 45 trials were discarded for reasons including trial inclusion of non-OA patients or non-MSC products or lack of randomization. The full articles of the remaining trials were assessed, and the trials were refiltered for eligibility, including only English language articles. Trials with insufficient control groups (16 trials) and of minimally manipulated cells (10 trials), or those meeting other criteria specified in Fig. 1, were excluded. It is important to note that we did not include trials of bone marrow aspirate concentrates, stromal vascular fractions or other minimally manipulated cellular sources, focusing only culture-expanded MSCs to consistently analyze the available clinical evidence, which represents a departure from previous systematic reviews [81–85].

Our approach resulted in 15 peer reviewed RCTs that we compared based on dosing, tissue source, MSC therapeutic product volume and carrier, clinical trial duration, and outcomes assessed by primary and secondary endpoints [56–71]. Overall, the 15 RCTs discussed in this section included patients who were treated with various doses of MSCs, ranging from 3.9 × 10^6^ to 150 × 10^6^ cells, or control treatments, including saline, PRP, HA, and others (Table 1). Of the RCTs analyzed in this section, 60% investigated MSCs from autologous sources, while the remaining 40% used allogeneic MSCs. Nearly half (7/15) of the RCTs involved bone marrow-derived MSCs (denoted MSC(M)); 6 of 15 used adipose tissue-derived MSCs (denoted MSC(AT)), and one study each used placenta tissue or Wharton’s jelly MSCs (MSC(WJ)). Although 15 RCTs were included, the total number of MSC-treated and control patients was small (610), making it difficult to render definitive conclusions regarding effect sizes, as has been noted in previous meta-analyses [76–80].

**Table 1:** Randomized control trials of culture-expanded mesenchymal stromal cells

Author	MSC Tissue Source	Cells/Dose (x10^6^)	Treatment Group Intervention	Control Group Intervention	Sample Size (Treatment/Control)	Male/Female	Baseline Pain Scores (VAS/WOMAC/KOOS)	Follow-up Duration (months)	Follow-up Evaluation
*Autologous*									
Lee et al. [57]	AT	100	sIA injection of MSCs in 3 mL of saline	sIA injection of 3 mL saline	24 12/12	6/18	MSC: 60/NA/NA Control: 56.4/NA/NA	0, 3, 6	WOMAC, VAS, KOOS, ROM, MRI (0, 6 months)
Freitag et al. [58]	AT	100	sIA injection of MSCs or 2 IA injections of MSCs at 0 and 6 months each suspended in 3 mL of saline	Ongoing conventional conservative management only	30 20/10	16/14	sIA MSC: NA/59.6/53 dIA MSC: NA/54.4/52.1 Control: NA/58.8/52.8	0, 1, 3, 6, 12	NPRS, KOOS, WOMAC, MOAKS (0, 12 months)
Lamo-Espinosa et al. [59]	M	100	sIA injection of MSCs suspended in 3 mL of Ringers Lactate solution followed by weekly injections of PRP (PRGF®), weekly, for 3 weeks (final volume of 8 mL)	sIA injection of PRP (PRGF®), weekly, for 3 weeks (final volume of 8 mL)	56 28/28	33/18	MSC: 5.3/33.4/NA Control: 5/31.9/NA	0, 3, 6, 12	VAS, WOMAC, WORMS (0, 12 months)
Lamo-Espinosa et al. [56, 60]	M	10, 100	sIA injection of low dose or high dose MSCs suspended in 1.5 or 3.0 mL of Ringers Lactate solution, respectively. Both followed by a sIA injection of 4 mL HA (Hyalone®)	sIA injection of 60 mg HA (Hyalone®) in a final volume of 4 mL	30 20/10	17/8	10-MSC: 7/37/NA 100-MSC: 6/28/NA Control: 5/29/NA	0, 3, 6, 12, 48	VAS, WOMAC, OARSI, WORMS (0, 6, 12 months)
Lu et al. [61]	AT	50	2 IA injections of MSCs at 0 and 3 weeks, SHAM injections at week 1 and 2, suspended in 2.5 mL	4 IA injections of 2.5 mL HA (week 0, 1, 2, and 3)	52 26/26	6/46	MSC: R 5.50, L 5.27/33.77/NA Control: R 4.96, L 4.92/32.15/NA	0, 6, 12	WOMAC, VAS, SF-36, MRI (0, 6, 12 months)
Emadedin et al. [63]	M	40	sIA injection of MSCs in 5 mL of saline	sIA injection of 5 mL saline	47 31/39	27/16	MSC: NA/NA/NA Control: NA/NA/NA	0, 3, 6	VAS, WOMAC, MCII, PASS
Bastos et al. [62]	M	40	sIA injection of MSCs in either 10 mL of PRP or PBS	sIA injection of corticosteroid (4 mg dexamethasone)	47 30/17	24/23	MSC: NA/NA/30.3 MSC-PRP: NA/NA/37.3 Control: NA/NA/36.9	0, 1, 2, 3, 6, 9, 12	KOOS, ROM
Wong et al. [64]	AT	14.6	HTO + Microfracture + sIA injection of MSC suspended in 2 mL of HA	HTO + Microfracture + sIA injection of 2 mL HA	56 28/28	29/27	MSC: NA/NA/NA Control: NA/NA/NA	0, 6, 12, 24	IKDC, Lysholm, Tegner, MOCART (0, 12 months)
Koh et al. [71]	AT	4.11	HTO + sIA injection of MSCs suspended in 3 mL of PRP	HTO + sIA injection of 3 mL PRP	52 26/26	11/33	Control: 45.4/NA/NA MSC: 44.3/NA/NA	0, 3, 12, 14–24	KOOS, Lysholm, VAS, Radiograph (0, 24 months)
*Allogeneic*									
Gupta et al. [65]	M	25, 50, 75, 150	sIA injection of MSCs suspended in 2 mL (25 & 50) or 4 mL (75 and 150) of PLASMA-LYTE A + 20 mg (2 mL) HA	sIA injection of either 2 or 4 mL PLASMA-LYTE A	60 40/20	15/45	25-MSC: 60.9/1315.8/NA 50-MSC:73.7/1498.4/NA Control-2: 61.0/1239.6/NA 75-MSC: 57.4/1470.6/NA 150-MSC: 46.6/1388.1/NA Control-4: 65.3/1382.0/NA	0, 1, 3, 6, 12	VAS, ICOAP, WOMAC Index MRI (0, 6, 12 months)
Chen & Hu et al. [66]	AT	16, 32, 64	sIA injection of MSCs suspended in 8 × 10^6^ cells/mL with cryoprotectant CryoStor® CS10	sIA injection of Hya Joint Plus 3 mL synovial fluid supplement	57 49/8	11/46	MSC: NA/NA/NA Control: NA/NA/NA	0, 1, 3, 6, 9, 12	WOMAC, VAS, KSCRS
Soltani et al. [67]	Placenta	50–60	sIA injection of MSCs suspended in 10 mL of culture media	sIA injection of 10 mL saline	20 10/10	2/18	MSC: NA/NA/NA Control: NA/NA/NA	0, 2, 6	VAS, KOOS, ROM, MRA (0, 6 months)
Vega et al. [68]	M	40	sIA injection of MSCs suspended in 8 mL of Ringer-lactate	sIA injection of 60 mg HA	30 15/15	11/19	MSC: 54/41/NA Control: 64/45/NA	0, 6, 12	VAS, WOMAC, Lequesne SF-12, MRI (0, 6, 12 months)
Matas et al. [69]	WJ	20	2 IA injection of MSCs at 0 and 6 months or 1 IA injections of MSCs at 0 months and IA injection of saline at 6 months	2 IA injection of HA at 0 and 6 months	29 21/8	11/18	sIA MSC: 44.8/37.4/NA dIA MSC: 39.4/35.6/NA Control: 38.7/28.9/NA	0, 1, 2, 3, 6, 9, 12	WOMAC, VAS, SF-36, WORMS (0, 6, 12 months)
Kuah et al. [70]	AT	3.9, 6.7	sIA injection of MSCs suspended in 2 mL	sIA injection of cell supernatant	20 16/4	12/8	3.9-MSC: 57/6.6/NA 6.7-MSC: 60.8/7.9/NA Control: 43.8/6.3/NA	0, 1, 3, 6, 9, 12	VAS, WOMAC, AQoL-4D, MRI (0, 12 months)

*M* bone marrow, *AT* adipose tissue, *WJ* Wharton’s jelly, *sIA* single intra-articular injection, *dIA* double intra-articular injection, *HA* hyaluronic acid, *PRP* platelet-rich plasma, *KOOS* knee injury and osteoarthritis outcome score, *VAS* visual analog scale, *ROM* range of motion, *MOAKS* MRI osteoarthritis knee score, *IKDC* International Knee Documentation Committee, *MRI* magnetic resonance imaging, *WOMAC* Western Ontario McMaster Universities Arthritis Index, *MOCART* magnetic resonance observation cartilage repair tissue, *MCII* minimally clinically important improvement, *PASS* patient acceptable symptom state, *KSCRS* new Knee Society Clinical Rating System, *WORMS* whole-organ magnetic resonance imaging score, *HTO* high tibial osteotomy, *PBS* phosphate-buffered saline, *MSC* mesenchymal stromal cell, *ICOAP* intermittent and constant pain score, *SF-36* 36-Item Short Form Survey, *SF-12* 12-Item Short Form Health Survey, *AQoL-4D* assessment of quality of life 4D questionnaire, *NPRS* numeric pain rating scale

Demographically, of the 610 treated patients, 356 patients (59%) were female, and 231 (38%) were male, while sex was not specified for 22 patients (3%). The average ages of patients per treatment group were generally between 50 and 60 years, with few outliers. Patients with knee OA as diagnosed by radiological and clinical evidence of OA [86] with Kellgren-Lawrence (KL) grades ranging from 1–4 were included in 13 of the 15 RCTs. Multiple clinically relevant endpoints, including the Western Ontario and McMaster Universities Arthritis Index (WOMAC), Visual Analog Scale (VAS) score, International Knee Documentation Committee (IKDC) score, Knee Injury and Osteoarthritis Outcome Score (KOOS), Numeric Pain Rating Scale (NPRS) score, and others, were used as patient-reported outcome measures (PROMs) and varied by study.

Overall, we observed a net positive treatment effect of MSCs, with clinical trials demonstrating improvement in VAS (9/12 trials), KOOS pain (4/5), WOMAC pain and total (9/11) or NPRS (1/1) scores relative to within-group baselines at their respective endpoints. There was considerable variation in the timing of endpoint collection among trials, including endpoint collection at 6 months (14/15 or 93%), 12 months (12/15 or 80%), and 24 months (3/15 or 20%). Overall, 12/15 RCTs showed improvements in most of their respective endpoints at final follow-up relative to baseline values, while relative to control groups, 11/15 trials found significantly better clinical outcomes. Our observations align with those of several meta-analyses that have reported positive effects of MSC treatments in OA [76–80].

### RCT patient populations

To further understand the differential responses to MSC treatments, we further investigated the heterogeneous OA patient populations enrolled in these trials. Some trials, including those of Freitag et al. [58] and Koh et al. [71], had a minimum age of 18 years, while Kuah et al. [70] and Matas et al. [69] restricted enrollment to patients aged 40–65 years old. Despite this variation and the limited sample sizes (average MSC treatment group *N* = 24.8), the mean ages for MSC treatment groups ranged from only 50.7 to 65.9 years across 14/15 studies, representative of an age range with a higher prevalence of OA [56–71]. Due to the higher prevalence of females with OA, we expected and observed a higher proportion of females (357, 59%) relative to males (231, 38%) treated. Furthermore, none of the studies, due to sample size limitations, included analyses based on sex or gender, which represent important variables that may influence treatment responses [87].

Fourteen out of fifteen RCTs reviewed in this section classified patients by KL grade, typically enrolling patients with KL grade 2–3 knee OA. However, within this classification, there is considerable heterogeneity in clinical phenotype (and disease endotype). We tried to parse differences in clinical phenotype looking at baseline pain scores (Table 1); 6/15 and 5/15 studies reported VAS and WOMAC scores, respectively. Other studies used the KOOS, Lysholm, Tegner, NPRS, IKDC and whole-organ magnetic resonance imaging score (WORMS). There were two studies that did not provide baseline values at all [63, 67] and one that provided values only as changes from baseline, which limits comparison between studies. Although most studies reported similar baseline scores, there was some variability that confounded the analysis of effective MSC dosing, as discussed below. Notably, two studies reported baseline VAS scores that varied by at least 10 mm between the treatment and control groups. Kuah et al. reported a baseline control VAS score of 43.8, much lower than the baselines for MSC groups of 57.0 and 60.8 for the 3.9 and 6.7 × 10^6^ dose groups, respectively [70]. The baseline VAS score in the Vega et al. study was 54 in the MSC group and 64 in the control group [70]. Clinical baseline phenotyping is clearly an important variable, and targeting patients with the right clinical phenotype and disease endotype will be key to successful future MSC clinical trials and outcomes in OA.

### MSC dose comparisons in RCTs

The studies included in RCT analyses used a range of doses from 3.9 × 10^6^ to 150 × 10^6^ cells. Dose optimization is not fully understood, with only 4/15 studies performing a dose escalation evaluation. Three of the four dose escalation groups received cells that were cryopreserved and subsequently thawed immediately prior to injection, while Lamo-Espinosa et al. used freshly cultured MSCs. Most of the studies showed positive effects of MSCs at moderate to high doses (>40 × 10^6^ MSCs), although as we discuss throughout this section, baseline patient phenotype and endotype, as well as other factors distinct among studies, may be confounding variables. Lamo-Espinosa et al. showed that only the higher dose of 100 × 10^6^ MSC(M), suspended in a 3 mL volume of Ringer’s lactate and coadministered with 4 mL of HA, was able to provide a significant improvement in the overall WOMAC score at 12 months. The moderate dose of 10 × 10^6^ MSC(M) suspended in 1.5 mL of Ringer’s lactate and coadministered with 4 mL of HA did not result in significant improvements in the WOMAC pain and function subscale scores. However, there was a significant improvement in the WOMAC stiffness subscale score at both 6 and 12 months. They also reported nonsignificant reductions in cartilage volume changes by magnetic resonance imaging (MRI) for the high-dose group at 12 months, while no changes were observed in the low-dose or HA groups, suggestive of possible chondroprotective effects of the higher doses of MSC(M), as discussed further in the next section [60].

Differential median baseline WOMAC total scores for the 10 × 10^6^ vs. 100 × 10^6^ dose reflective of variations in patient clinical phenotype and endotype might have contributed to the differential efficacy of the MSC doses [60]. This concept is supported by the findings of the study by Kuah et al. [70], in which patients with lower (albeit not statistically significant) baseline VAS scores were randomized to the group receiving a lower dose (3.9 × 10^6^) of MSC(AT), which showed good efficacy, while patients with lower baseline pain scores in the Lamo-Espinosa et al. trial were randomized to the group receiving a high dose (100 × 10^6^) of MSC(M), which showed better efficacy than the moderate dose (10 × 10^6^) at the 12-month endpoint [60].

Lamo-Espinosa et al. conducted a subsequent 4-year follow-up study with 27 of the 30 patients [56]. Both the high (100 × 10^6^) and moderate (10 × 10^6^) MSC(M) dose groups continued to show significant differences according to VAS scores relative to the control group. Strikingly, overall WOMAC scores were significantly improved in the moderate- but not high-dose MSC(M) group relative to the control group at 48 months. The control group treated with HA alone showed short-term improvements in WOMAC pain, function, and overall scores at 3 and 6 months, but not at longer follow-up timepoints of 12 or 48 months. The observed differences in WOMAC scores at 48 months between the high- and moderate-dose groups suggest the need for longer follow-ups and additional baseline patient clinical phenotyping and endotyping (discussed in the *Roadmap* section) to understand the differences in personalized disease trajectories in the high- vs. moderate-dose groups. Interestingly, the moderate-dose group with more baseline pain (higher WOMAC scores) showed better WOMAC scores than the control group at the 48-month timepoint.

Similarly, Chen and Hu et al. demonstrated improvements in overall WOMAC scores at 48 weeks posttreatment in the group receiving the highest dose (64 × 10^6^) of allogeneic MSC(AT) relative to HA controls; all dose groups (16 × 10^6^, 32 × 10^6^, and 64 × 10^6^) showed improvements in WOMAC total scores and stiffness, pain, and function subscores from baseline to 48 weeks posttreatment [66]. The Knee Society Clinical Rating System (KSCRS) symptom score was significantly different at week 48 in the 64 × 10^6^ high-dose group vs. the HA control group. Interestingly, the moderate dose group (16 × 10^6^) had significantly higher (less severe) baseline KSCRS patient satisfaction scores, suggestive again of baseline clinical phenotype effects of patient selection on observed treatment outcomes.

Conversely, two studies, Gupta et al. [65] and Kuah et al. [70] showed more efficacious outcomes at lower MSC doses. Gupta et al. included 25 × 10^6^, 50 × 10^6^, 75 × 10^6^, and 150 × 10^6^ pooled bone marrow allogeneic MSCs (Stempeucel®) combined with HA injections. Although the study was not adequately powered for definitive dose comparisons (*N* = 10 patients/dose group, *N* = 20 for control group), the authors observed a trend toward pain reduction measured by VAS, WOMAC and Intermittent and Constant Pain Score (ICOAP) scoring criteria in the low-dose group (25 × 10^6^ cells); this was not statistically significant when compared to control (Plasma-Lyte A) [45]. Importantly, Gupta et al. included MSC(M) derived from pooled allogeneic donors (*N* = 3), and there is clinical evidence in patients with graft-versus-host disease (GVHD) that pooled bone marrow MSCs have greater efficacy than single-donor bone marrow MSCs [88].

Kuah et al. [70], similar to Gupta et al. [65], showed that cryopreserved allogeneic MSC(AT) delivered with cell supernatant were efficacious at lower doses of 3.9 × 10^6^ and 6.7 × 10^6^ in significantly improving WOMAC pain subscale scores at 6 months, but this effect was sustained at 12 months only in the low-dose (3.9 × 10^6^) group [70]. However, none of the WOMAC subscale scores were significantly different between the control (culture supernatant) and MSC(AT) groups at the 12-month timepoint. Kuah et al. used MSC(AT) from individual, nonpooled healthy donors, different from the Gupta et al. studies with pooled MSC(M). Furthermore, the tissue sources differed between the two studies. Additionally, relative to all the MSC(M) treatment groups in the study by Gupta et al. (except their 150 × 10^6^ MSC(M) group), the patients in the Kuah et al. study in the 3.9 × 10^6^ and 6.7 × 10^6^ dose groups had lower mean baseline VAS pain scores of 57.0 and 60.8, respectively. The 25 × 10^6^, 50 × 10^6^, 75 × 10^6^ and 150 × 10^6^ dose groups in the Gupta et al. study had mean baseline VAS scores of 67.0, 78.8, 71.3, and 62.0, respectively. Thus, MSC dosing needs to be viewed in the context of baseline patient clinical phenotype and, as we discuss later, disease endotype. Interestingly, Kuah et al. randomized patients with lower (albeit not statistically significant) baseline VAS scores to the lower dose (3.9 × 10^6^) MSC(AT) group, while Lamo-Espinosa et al. randomized patients with lower baseline pain scores (based on the WOMAC) to the high dose (100 × 10^6^) MSC(M) group [60]. In both cases, the patients with the lower baseline clinical phenotype responded more efficaciously to MSCs, irrespective of dosing.

Taken together, the limited RCT data on dose escalations provide no evidence of a clear winner in terms of patient-reported outcomes. While both the Gupta et al. [65] and Kuah et al. [70] groups showed that lower doses of allogeneic MSCs were better (although neither were statistically significant vs. controls at 12 months), Lamo-Espinosa et al. [60], Chen and Hu et al. [66] concluded that higher allogeneic MSC(M) or MSC(AT) doses were better, showing statistically significant differences in WOMAC scores at 12 months. However, the longer 48-month follow-up by Lamo-Espinosa et al. [56] did not show sustained improvements in PROMs in the higher 100 × 10^6^ MSC(M) group. Furthermore, cartilage volume losses at 12 months (discussed in detail below) did not change at the higher doses but were reduced at the lower dose and in the control HA group [60], suggestive of greater chondroprotective effects at higher MSC doses. Two meta-analyses on the use of MSCs in OA (albeit including a group receiving a mixture of culture-expanded MSCs and minimally manipulated cells) found that medium to higher doses were more effective and cited 40 × 10^6^ cells [89] or 50 × 10^6^ cells [81] as the cell threshold for significant patient-reported benefits. Our nonstatistical analyses are supportive of moderate to higher MSC doses, but we note that the effects of baseline patient pain scores, differences between pooled versus single allogeneic donors, differences in MSC tissue sources, the use of MSC therapies in combination with other bioactive agents, and other variables confound the effects of MSC dosing. Matching the clinical phenotype (and disease endotype of OA patients) to MSC basal fitness seems to be key to achieving successful clinical outcomes.

### Repeat MSC injection RCTs

There are only three published RCTs in which repeat MSC intra-articular injections were administered. Freitag et al. used cryopreserved autologous MSC(AT) at doses of 100 × 10^6^ cells suspended in 3 mL saline [58]; Matas et al. opted for cryopreserved 20 × 10^6^ allogeneic MSC(WJ) [69]; and Lu et al. used cryopreserved 50 × 10^6^ autologous MSC(AT). Matas et al. and Freitag et al. designed their trials to be run with three treatment groups receiving either a single or double intra-articular MSC injections (6 months apart) vs. a control group. Lu et al. injected 50 × 10^6^ MSC(AT) at 0 and 3 weeks along with sham injections at weeks 1 and 2, which were compared to 2.5 mL HA injections at weeks 0, 1, 2, and 3.

Interestingly, Freitag’s group initially included a fourth arm of 40 × 10^6^ MSC(AT) injected at higher monthly frequencies (1, 2, 3 and 6 months) that was scrubbed due to emerging data showing injection-site pain with frequent monthly injections [75]. Although there were significant improvements for both treatment groups (single injection; *N* = 10, double injection; *N* = 10) compared with the control group (conservative management; *N* = 10), there was no significant difference between the single- and double-injection groups at least in KOOS and WOMAC scores at the 12-month completion of the study. A combined metric of KOOS, WOMAC, and NPRS scores was used to calculate the percentage of participants achieving a minimal clinically important difference at 12 months relative to baseline values, and values of 25.7%, 84.1%, and 87.1% were found for the control, single, and double injection groups, respectively. Notably, while there were no differences in KOOS and WOMAC scores at 12 months between the single- and double-injection groups, there was a reduction in cartilage volume losses in the double- vs. single-injection groups. Interestingly, as in this example, we have noticed a pattern in which structural changes and PROMs tend to occur independently, with improvements in only one or the other occurring at a time.

Conversely, the design of the Matas et al. trial included a control group (*N* = 8) that also received repeat dosing of intra-articular HA, while the single injection MSC(WJ) group (*N* = 9) received a control second injection at 6 months. The double injection MSC(WJ) group (*N* = 9) received 20 × 10^6^ cells in 3 mL of saline at both baseline and 6 months postinjection. Using both the WOMAC total and pain scores, significant improvements were observed in the double injection MSC(WJ)-treated vs. HA groups. This is especially consequential because the HA control group also received a second injection at 6 months that boosted the previously waning WOMAC pain and function subscale score improvements [69]. The single injection MSC(WJ)-treated group showed continued improvements up to 9 months, but WOMAC scores reverted toward the levels of the HA control group (after the second HA injection) at the 12-month follow-up. Longer follow-up times would have afforded a better evaluation of the synergistic or additive effects of the double MSC(WJ) injections. Interestingly, Matas et al. did not report changes in cartilage volume with single vs. dual injections.

The Lu et al. trial employed a unique dosing method in which four injections were given a week apart for both the MSC and control groups. The control group received four 2.5 mL injections of HA, while the treatment group received 50 × 10^6^ MSC(AT) during the first and third injections and 2.5 mL of HA in the second and final weeks. During both the 6- and 12-month follow-ups, there were significant improvements in VAS pain scores relative to the HA control group and significantly increased cartilage volumes relative to baseline in terms of modified WORMS, while the HA control group showed cartilage volume losses at 12 months [61].

Taken together, all three trials reported benefits of dual injections, although only the Matas and Lu trials showed differences in PROMs at 12 months, while the Freitag and Lu trials showed gains in cartilage volume or a lack of cartilage volume losses at 12 months with dual injections.

### Autologous vs. allogeneic MSCs in RCTs

RCTs investigating autologous MSCs made up the majority (9/15) of the studies reviewed, while the remaining (6/15) studies investigated MSCs of allogeneic origin. The autologous tissue of origin was split between bone marrow (5/9) and adipose tissue (4/9), while allogeneic sources were split between adipose tissue (2/6) and bone marrow (2/6). The remaining allogeneic trials used MSCs from Wharton’s jelly (1/6) or the placenta (1/6). Most of the dose escalation trials (3/4) used allogeneic MSC sources, while the three repeat injection trials were divided two-to-one between autologous and allogeneic MSCs [60, 65, 66, 68–70, 75].

In 5/6 of the MSC allogeneic trials, there was a significant improvement in PROMs (WOMAC, KOOS, and KSCRS scores) relative to the control groups. This was true regardless of doses ranging as low as 3.9 × 10^6^ MSC(AT) [70] and as high as 64 × 10^6^ MSC(M) [70]. The exception was the Gupta et al. [65] trial using cryopreserved pooled MSC(M) doses combined with HA injection, which were unable to achieve a significant difference from the control treatments at 12 months in terms of VAS or WOMAC scores [65]. However, the lowest dose was most efficacious in reducing pain scores. Taken together, 5/6 allogeneic MSC trials reported positive patient outcome effects at low to moderate doses (3.9–40 × 10^6^ MSCs) [66–70].

Similarly, in 8/9 RCTs using autologous MSCs, there was significant improvement in PROMs [56–62, 71]. Typically, the autologous studies used doses from 10–100 × 10^6^ MSCs, including the studies of Bastos et al. [62], Wong et al. [64], and Lamo-Espinosa et al. [56, 60]. The exception was the trial of Koh et al., who performed high tibial osteotomy in addition to administering low-dose (4 × 10^6^) MSC(AT) and 3 mL of PRP, which resulted in significant improvement in VAS pain scores relative to the high tibial osteotomy and PRP alone control group [71]. Two studies used saline as a control [57, 63]. The use of saline as a control necessitated an earlier 6-month endpoint. Emadedin et al. showed a mean change in overall WOMAC scores of >10 points in the MSC(M) vs. saline groups at 6 months [63]. Lee et al. showed significant improvements in WOMAC scores at 6 months in the MSC(AT) group but not the saline control group [57]. Freitag et al. showed that repeat moderate-dose (40 × 10^6^) autologous MSC(AT) injections improved WOMAC total scores relative to conservative management [58]. Taken together, from the limited dataset on RCTs, moderate to higher doses (10–100 × 10^6^) of autologous MSCs (except for in the study of Koh et al., who used 4 × 10^6^ autologous MSC(AT) in combination with PRP and surgical intervention) resulted in positive PROMs at the study endpoints. These findings suggest that although the efficacy of MSCs has been demonstrated relative to the current standard of care at various cell doses, there is no definitive dose range that has been reproducibly and consistently shown to be efficacious; multiple factors, including OA patient clinical phenotype, molecular endotype, and MSC basal fitness, need to be considered, matched and optimized.

In summary, the 15 RCTs with 610 treated patients showed positive effects of MSCs relative to control treatments at 12-month timepoints in terms of PROMs. However, clear distinctions are not apparent in regard to dose dependencies, MSC tissue of origin, and autologous or allogeneic MSC sources. We did note that in 7/9 autologous MSC trials, moderate to high (10 × 10^6^—100 × 10^6^) MSC doses were used, while 5/6 allogeneic MSC trials used low to moderate doses (4 × 10^6^—40 × 10^6^), suggesting possible augmented basal fitness of allogeneic-, nonpatient-derived MSCs. However, culture parameters to expand MSCs and cryopreservation/thawing can further affect basal fitness, as previously reported [90, 91]. Additionally, we noted variations in baseline VAS or WOMAC scores across the 15 RCTs and between treatment groups within a given trial that were suggestive of differences in the clinical phenotype of patients that further confound MSC dosing effects. All clinical trials reported PROMs, while 10/15 reported effects on cartilage repair, and a few reported changes in local and systemic biomarkers (3/15) [62, 66, 70]. The mechanisms through which MSCs may exert these observed changes are discussed in the next section.

## MSC mechanisms of action in knee OA

Despite the completion of 15 peer-reviewed RCTs, there is limited evidence on the clinical mechanism of action of MSCs in OA. An improved understanding of the mechanism of action would be beneficial in the following ways: (i) it would facilitate the design of improved MSC therapies with properties tailored to achieve the desired effects in OA; (ii) it would facilitate the selection of critical quality attributes (CQAs) for MSC investigational products that are relevant to the mechanism of action and could be used as potential release criteria; (iii) in patient stratification or precision medicine-based approaches, it could facilitate the selection of patients with a higher response likelihood to MSC interventions based on disease status biomarkers relevant to the therapeutic mechanism of action; and (iv) it would facilitate the selection of relevant readouts or biomarkers that inform clinical responses based on the expected clinical effects of an MSC intervention.

To underscore the importance of understanding the mechanism of action, a previous biologic license application for Remestemcel-L™, an allogeneic MSC(M) product for the treatment of steroid-refractory acute GVHD, was rejected by the United States Food and Drug Administration (FDA), partially due to the inadequacy of the CQAs employed by the sponsor; the researchers did not demonstrate a clear relationship of the MSC CQAs to the clinical potency of the product [92]. The FDA statement also noted that while the assays used as CQAs—MSC(M) expression of TNF receptor 1 protein and MSC(M)-mediated inhibition of IL-2Rα expression in activated lymphocytes—were aligned with the hypothesized immunomodulatory mechanism of action for the MSC(M) product, there was a lack of clinical evidence submitted to support this mechanism [92]. The FDA recommended using a multivariate matrix of CQAs, as we have recently proposed [92]. Altogether, while the mechanisms may be complex and multimodal for MSC therapy, particularly in the context of OA, understanding these mechanisms is key for obtaining regulatory authorization and for eventual refinement of MSC therapies.

The following subsections provide an overview of current insights into MSC mechanisms of action in OA, with emphasis on immunomodulatory mechanisms. We provide an overview of in vitro and preclinical studies that provide insights into MSC-mediated immunomodulation as an important mechanism of action in OA. We further detail the clinical evidence (beyond the 15 RCTs in the previous section) where available, discussing immunomodulatory, chondroprotective, regenerative and other effects of MSCs in the treatment of knee OA.

### MSC anti-inflammatory and immunomodulatory effects on OA

MSC-mediated anti-inflammatory and immunomodulatory effects on OA inflammation are also known to play a central role in OA, with both innate and adaptive mediators involved in the pathophysiology [93]. In particular, the synovium, consisting primarily of fibroblast-like synoviocytes and MΦs, becomes inflamed in OA; neovascularization as well as a leaky endothelium within the subintimal layer allows increased cellular and molecular infiltration within the synovium and synovial fluid [94]. Lineage-tracing experiments in a rheumatoid arthritis mouse model have also indicated the presence of a self-renewing population of resident synovial MΦs that may represent a source of additional immune cells in the synovium [95]. The infrapatellar fat pad within the knee joint is also highly vascularized and contributes to increased levels of inflammatory cells and mediators [96]. MΦs produce inflammatory and catabolic mediators in OA, and our group has shown that MΦs represent the most abundant leukocyte population within the synovial fluid of OA patients [97]. Indeed, CD14^+^CD16^-^ (classical) and CD14^+^CD16^+^ (intermediate) MΦ subpopulations were found to be positively and negatively correlated with more severe PROMs in a cohort of knee OA patients [97]. The synovial membrane in OA contains several other immune populations, including T and B cells, natural killer cells, neutrophils, dendritic cells, mast cells, and granulocytes, that play roles in OA pathology [97]. In particular, T cells are relatively abundant within the OA synovium and synovial fluid, and altered profiles of T-cell subsets, including Th1, Th17, and Treg cells, have been documented [98]. We have further shown that activated T-cell subsets correlate with different MΦ subsets [97]. Anti-inflammatory and immunosuppressive effects of MSCs on MΦs and T cells have been widely documented [99, 100] and thus represent plausible cellular effectors of intra-articular injections of MSCs in OA.

Evidence of the anti-inflammatory and immunomodulatory functionality of MSCs has been extensively reviewed in various preclinical studies [72, 101]; nonetheless, we highlight some of the key findings here to provide context for immunomodulation as a primary MSC mechanism of action. Notably, work by Fahy et al. demonstrated that conditioned medium derived from human OA synovial macrophage cultures inhibited chondrogenic differentiation of MSC(M) [102]. In vitro polarization of human peripheral blood-derived monocytes to proinflammatory subtypes had similar effects on inhibiting MSC(M) chondrogenic differentiation, suggestive of a potential proinflammatory blockade of chondrogenic differentiation in an OA joint. The presence of IL-6, CCL18 and other proinflammatory factors in synovial macrophage conditioned medium was thought to inhibit MSC(M) chondrogenic differentiation. Thus, addressing proinflammatory imbalance in the joint may be a prerequisite to enabling endogenous chondroprogenitor differentiation and proliferation repair mechanisms. In vitro, MSC(AT) showed anti-inflammatory effects on OA chondrocytes and synoviocytes harvested from the femoral condyle of OA patients undergoing total knee replacement [103]. Indirect cocultures of MSC(AT) with OA chondrocytes demonstrated reduced hypertrophy and promoted a mature differentiated chondrocyte phenotype, while more modest anti-inflammatory effects were observed with MSC(AT)-conditioned medium, suggesting the importance of cross-talk between MSC(AT) and chondrocytes in mediating these effects. These data support the hypothesis that MSCs enable chondrocyte repair and tissue remodeling in part by alleviating anti-inflammatory cues that inhibit chondrocyte proliferation and appropriate differentiation.

The hypothesis that immunomodulation and inflammation play a central role in MSC-mediated OA repair and tissue remodeling is further supported by data from our laboratory showing that iron nanoparticle-labeled syngeneic murine MSC(M) injected into immunocompetent C57Bl/6 mice with surgically induced OA were retained within the synovium up to 4 weeks postsurgery; importantly, the presence of the iron-labeled MSC(M) was confirmed with dual Prussian Blue and SCA-1^+^ (a murine MSC marker) cell staining by immunohistochemistry [104]. We additionally showed that these MSC(M) were apoptotic and phagocytosed by CD206^+^ MΦs, which resulted in an increase in the CD206:iNOS-positive MΦ ratio in the MSC(M)- vs. saline-treated mice [104]. Our data are highly supportive of an anti-inflammatory effect of MSC(M) and interactions with local MΦs in the articular joint to modulate them to more pro-resolving subtypes. Interestingly, Schelbergen et al. showed in mouse models that the reparative effects of MSC(AT) were limited to inflammatory models of OA, namely, collagenase-induced OA (CIOA), but not in the post-traumatic surgical injury and destabilization of the medial meniscus (DMM) model [105]. Additionally, they found that MSC(AT) were most effective in mice with high synovitis scores and systemic inflammation, as evidenced by serum levels of S100A8/9. A follow-up study by van Dalen et al. showed that polymorphonuclear cells (PMNs) form clusters around MSC(AT) injected in the CIOA mouse joint and that this may be mediated by IL-1β priming, which upregulates MSC(AT) expression of chemotactic factors and enhances PMN phagocytic activity in coculture experiments [106]. These observations challenge the utility of the well-accepted DMM model for measuring MSC-mediated cartilage reparative effects and support the important role of the immunomodulatory mechanisms of MSCs in OA models.

**Fig. 2:**
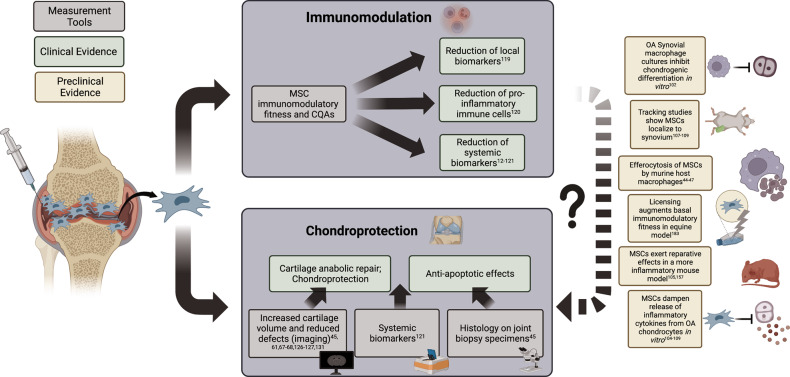
Clinical and preclinical evidence of mesenchymal stromal cell mechanisms of action on immunomodulation and chondroprotection in knee osteoarthritis. Solid arrows are used to demonstrate mechanisms for which there is supporting clinical evidence: dashed arrows indicate the relationship between immunomodulatory and chondroprotective mechanisms supported by preclinical evidence. MSC mesenchymal stromal cell, CQAs critical quality attributes, OA osteoarthritis

Cell tracking studies in equine [107] and ovine [108, 109] OA models demonstrate that autologous and allogeneic MSCs can persist within the joint for the duration of study periods ranging from 8–14 weeks postinjection. Notably, these studies have shown that injected MSCs home to the synovium rather than to cartilage [107–109], supporting MSC modulation of synovial inflammation as a primary therapeutic target in OA. Feng et al. [109] used allogeneic MSC(AT) labeled with superparamagnetic iron oxide (SPIO) combined with HA to observe MSC localization primarily to the synovium, particularly around synovial CD68^+^ macrophages. In this ovine OA model, the levels of the inflammatory factors TNF-α and IL-6 in the synovial fluid of the MSC(AT) + HA-treated group were significantly lower than those in the control groups; concomitantly, these groups also showed significantly thicker cartilage of the tibial plateau relative to controls [109]. Barrachina et al. [110] used an equine model to show that MSC(M) in chemically (amphotericin)-induced OA significantly reduced inflammatory markers via histopathological analysis of the synovium at both 2 and 6 months. Subsequently, both unprimed MSC(M) and MSC(M) primed with IFNy and TNF-α (to enhance the basal immunomodulatory fitness of the cells) resulted in delayed cartilage degeneration. However, the MSC(M)-primed group showed an anabolic response via an upregulation of collagen type II (COL2), aggrecan (ACAN) and cartilage oligomeric matrix protein (COMP) gene expression. Although both MSC(M) treatment groups showed similar macroscopic cartilage appearances, the differential gene expression suggests higher quality tissue remodeling due to MSC priming with proinflammatory cytokines, supportive of primary MSC immunomodulatory functionality. Moreover, canine MSC(AT) genetically modified to overexpress the pleiotropic growth factor platelet-derived growth factor (PDGF) or antioxidant factor heme-oxygenase 1 (HO-1) showed improvements in pain and lameness scores in PDGF-MSC(AT)-treated but not HO-1 MSC(AT)-treated beagles with cranial cruciate ligament transection, which mimics posttraumatic OA [111]. Notably, the expression of tissue inhibitor of metalloproteinases was significantly upregulated, effectively reducing catabolic MMP-13 and nerve growth factor. Furthermore, an increase in collagen type 2 was observed in canine chondrocytes [112]. Taken together, the in vitro and preclinical data support the chondroprotective and tissue remodeling effects mediated by MSCs in parallel to or secondary to primary immunomodulatory and anti-inflammatory effects, visualized in Fig. 2.

Now, it should be noted that the role of inflammation in OA is not fully understood, and it is unclear whether inflammation represents a primary etiology of the disease or whether it arises secondary to other processes, such as joint damage and altered mechanical signaling within the cartilage [113]. Moreover, the inflammatory landscape of OA appears to be distinct from that of other arthritides, and the levels of classical Th1 cytokines such as TNF-α and IL-1, which are prevalent in RA, are present at very low levels in the synovial fluid of OA patients [114]. Indeed, the level of inflammation in OA may vary temporally, and it has been posited as a distinguishing feature for OA patient endotype, as discussed below [115]. In the context of MSC therapies, an improved understanding of inflammatory features and their temporal kinetics may be crucial given that inflammatory priming is critical to induce immunomodulatory functions in MSCs [116]. Thus, for MSCs to achieve therapeutic benefit in OA via immunomodulation, it may be important to better delineate inflammatory patient endotypes and/or inflammatory flares using sensitive imaging and/or biomarker evaluations that would enable improved patient selection based on responder likelihood to MSC therapy.

### Clinical evidence of MSC immunomodulation in OA

While the findings of preclinical studies (extensively reviewed [117, 118]) support MSC-mediated immunomodulation in OA as an important mechanism of action, clinical evidence has thus far been limited. In a twelve-patient Phase I/IIa knee OA clinical trial with autologous MSC(M) injections, our group provided the first clinical evidence to demonstrate that MSCs modulate inflammation within the OA joint [119]. We observed a significant reduction in synovial fluid levels of the proinflammatory cytokine IL12p40 at 3 months relative to baseline along with a trend toward reduced levels of the intermediate CD14^+^CD16^+^ MΦs [119]. In parallel, we noted significant improvements in KOOS pain, symptom, and quality of life and WOMAC stiffness subscale scores relative to baseline [119].

Systemic and local levels of inflammatory biomarkers after intra-articular MSC injections have also been investigated in recent knee OA trials. A study by Li et al. demonstrated systemic anti-inflammatory effects of MSC(M) injections and platelet lysate (three monthly injections with MSCs and platelet lysate injected three days apart) relative to arthroscopic debridement and HA injections in knee OA patients (KL grade 0–2, *N* = 40–46/group); significant reductions in serum TNF-α and IL-6 were noted within the MSC(M)-treated group at 6 and 12 months relative to baseline and to the HA controls [120]. In a three-patient study, Sadri et al. also reported a trend toward decreased serum IL-6 and transiently increased IL-10 in knee OA patients (KL grade 2–3) receiving allogeneic MSC(AT), albeit with a limited sample size and no statistical testing [121].

In contrast to the above, Bastos et al. have shown that there were no significant differences in synovial fluid levels of various inflammatory cytokines (IL-17A, IFN-γ, TNF -α, IL-10, IL-6, IL-4, and IL-2) between knee OA (KL grade 1–4) patient groups treated with autologous MSC(M) (40 × 10^6^ fresh cells), autologous MSC(M) plus autologous PRP, or corticosteroids at 6 and 12 months after treatment (*N* = 10–13/group) [62]. Notably, the authors detected significant reductions in the levels of the anti-inflammatory cytokine IL-10 at 12 months relative to baseline in all three groups, which the authors postulated may be related to a resolution of inflammation across groups due to a potential compensatory role of IL-10 in the inflammatory context of OA [62]. Nonetheless, the MSC injections may have effected immunomodulatory changes at earlier timepoints that were missed upon synovial fluid collection at 6 months. Indeed, in our previous knee OA clinical trial with autologous MSC(M), we probed inflammatory changes within the synovial fluid at baseline and 3 months to detect earlier changes in joint inflammation that coincided with maximal improvements in patient pain, symptoms and function [119].

### Clinical evidence of chondroprotection and cartilage repair

To date, most OA clinical trials investigating disease modification by MSC therapeutics have focused on cartilage changes, given that cartilage degradation is a hallmark of OA, as well as a history of regulatory bodies emphasizing the need for structural changes to achieve therapeutic effects in OA [122]. Altogether, we identified 21 clinical studies (Table 2) that provided evidence of chondroprotective or cartilage regenerative effects of intra-articular MSC injections in knee OA, including through imaging modalities, second-look arthroscopy, analysis of cartilage tissue biopsies, and the measurement of biomarkers for cartilage breakdown in serum, plasma and urine. While these effects are promising, it should be noted that these observations provide little insight into the true mechanisms of action given that MSCs can modulate the joint microenvironment through interactions with a variety of cell types within the joint, including chondrocytes and other cells. As noted above, these effects may occur downstream of MSC-mediated immunomodulation in the OA joint, as demonstrated by Fahy et al. and others in vitro [110, 117, 118]; further research is needed to delineate the interrelationships among these mechanisms of action. While most published studies investigating cartilage repair have suggested regenerative (14/21 or 66%) or chondroprotective (4/21 or 20%) effects of MSC injections, several others (3/21 or 14%) have not detected improvements in cartilage volume or integrity. Moreover, a meta-analysis of five published clinical studies reported that the quality of evidence for MSC-mediated chondroprotection or regeneration was limited, pointing to the need for additional higher powered and well-controlled studies [83]. Insights into chondroprotection and regeneration have primarily relied on MRI measurements, which are associated with several limitations, including a lack of sensitivity and a standardized methodology for MRI measurements and analyses. Importantly, previous work has shown that the relationship between cartilage structural changes and patient pain or other symptoms is complex and not necessarily correlative (reviewed in [123–125]), suggesting that chondroprotection and regenerative effects may not map onto patient clinical responses. Nonetheless, we review clinical studies evaluating the regenerative and chondroprotective effects of intra-articular MSC injections, as these provide useful evidence for the potential disease-modifying effects of MSCs within the OA joint.

**Table 2:** Clinical effects of culture-expanded mesenchymal stromal cell injections on knee OA cartilage

Author	MSC tissue source	Cell dose (x10^6^ cells)	Treatment group intervention	Comparison (baseline or control)	Number treated	Cartilage outcome measures	Time points	Cartilage outcomes
*Autologous*								
Jo et al. [45]	AT	10, 50, 100	sIA injection of MSC	Baseline	12 (100 × 10^6^ dose); 3 (50 × 10^6^ dose); 3 (10 × 10^6^ dose)	MRI (volume, defect size), Arthroscopy, Histology (Col I, Col II, SafO), X-ray (KL, JSW)	0, 3, 6 months (MRI, X-ray); 0, 6 months (arthroscopy, histology)	Regeneration favouring high-dose group by MRI, arthroscopy, histology
Lamo-Espinosa et al. [60]	M	10, 100	sIA injection of MSC + 60 mg HA	Baseline, 60 mg HA control	10 (100 × 10^6^ dose); 10 (10 × 10^6^ dose); 10 (control)	MRI (WORMS), X-ray (JSW)	0, 6, 12 months	Possible regeneration favouring high-dose group by X-ray and MRI
Freitag et al. [58]	AT	100	sIA injection of MSC ± second injection at 6 months	Baseline, conservative management control, and dose groups	10 (single dose); 10 (two doses); 10 (control)	MRI (MOAKS)	0, 12 months	Chondroprotection favouring two-dose group
Lamo-Espinosa et al. [59]	M	100	IA injections of MSC at 0, 1, and 2 weeks + 8 mL PRP	Baseline, PRP only control	30 (MSC+PRP); 30 (control)	MRI (WORMS), X-ray (JSW)	0, 12 months	No significant effects
Lee et al. [57]	AT	100	sIA injection of MSC	Baseline, 3 mL saline (vehicle) control	12 (MSC group); 12 (control)	MRI (volume, defect size), X-ray (KL, JSW, HKA)	0, 6 months	Chondroprotection; significant increase in defect size observed in saline- but not MSC-treated group by MRI
Song et al. [126]	AT	10, 20, 50	Bilateral IA injections of MSC at 0, 3 weeks ± third injection at 48 weeks	Baseline	6/dose group	MRI (volume)	0, 12, 24, 48, 72, 96 weeks	Regeneration favouring high-dose group with subsequent reduction in cartilage volume at 96 weeks
Chahal et al. [119]	M	1, 10, 50	sIA injection of MSC	Baseline	4 (50 × 10^6^ dose); 4 (10 × 10^6^ dose); 4 (1 × 10^6^ dose)	MRI (T2, WORMS), ELISA (HA, COMP, C1–2C, C2C, CTXII)	0, 6, 12 months (MRI); 2, 6, 12, 24, and 48 week (ELISA)	Possible chondroprotection; no changes by MRI but catabolic biomarkers were significantly reduced and favoured high-dose group
Chen et al. [127]	AT	50	sIA injection of MSC	Baseline	12	MRI (MOAKS), X-ray (KL, JSW)	0, 48 weeks	Regeneration indicated by reduced MOAKs articular cartilage pathology scores
Lu et al. [61]	AT	50	Bilateral IA injections at 0 and 3 weeks	Baseline, 25 mg HA control	26/group	MRI (volume)	0, 24, 48 weeks	Regeneration in MSC group with degeneration in HA group
Orozco et al. [128]	M	40	sIA injection of MSC	Baseline	12	MRI (T2, PCI)	0, 6, 12 months	Regeneration indicated by significant reduction in PCI scores at 6 and 12 months
Rich et al. [129]	M	40	sIA injection of MSC	Baseline	50	MRI (T2, PCI)	0, 12 months	Regeneration indicated by significant reduction in T2 values
Soler et al. [130]	M	40	sIA injection of MSC	Baseline	15	MRI (T2, PCI)	0, 6, 12 months	Regeneration indicated by significant reduction in T2 values at 6 and 12 months
Al-Najar et al. [131]	M	30.5	IA injection of MSC at 0 and 1 month	Baseline	13	MRI (thickness)	0, 6, 12 months	Regeneration indicated by increased cartilage thickness at 12 months
*Allogeneic*								
Gupta et al. [65]	M	25, 50, 75, 150	sIA injection of MSC + 20 mg HA	Baseline, vehicle + HA control	10 (for each MSC dose); 20 (control)	MRI (WORMS), X-ray (unspecified parameters)	0, 6, 12 months (MRI); 0, 3, 6 months (X-ray)	No significant effects
Sadri et al. [121]	AT	100	sIA injection of MSC	Baseline	3	MRI (thickness, volume), ELISA (COMP, HA, CTX-II, MMP-3)	0, 6 months (MRI); 0, 1 week, 1, 3, 6 months	Possible chondroprotection and regeneration by MRI and ELISA but small sample size
Soltani et al. [67]	Placenta	50–60	sIA injection of MSC	Baseline, saline control	10 (MSC); 10 (control)	MRA (thickness)	0, 24 weeks	Regeneration indicated by increased cartilage thickness relative to baseline in MSC but not control group
Zhao et al. [132] and Lu et al. [133]	AT	10, 20, 50	Bilateral IA injections at 0 and 3 weeks	Baseline	7 (10 × 10^6^ group); 8 (20 × 10^6^ group); 7 (50 × 10^6^ group)	MRI (volume, WORMS, T1_rho_, T2, T2star, R2 star, ADC, FA)	0, 48 weeks	Regeneration favouring high-dose group
Vega et al. [68]	M	40	sIA injection of MSC	Baseline, 60 mg HA control	15 (MSC group); 15 (control)	MRI (T2, PCI)	0, 6, 12 months	Regeneration indicated by improved PCI in MSC(M)-treated patients relative to HA at 12 months
Matas et al. [69]	WJ	20	sIA injection of MSC ± second injection at 6 months	Baseline, 60 mg HA control	10 (single dose); 10 (two doses); 8 (control)	MRI (WORMS)	0, 6, 12 months	No significant effects
Park et al. [134]	CB	11.5–20	Implantation of HA hydrogel containing MSC(WJ)	Baseline	6	MRI (dGEMRIC ΔR1), Arthroscopy, Histology (Col II, SafO)	0, 3 years (MRI); 0, 12 weeks, 12 months (arthroscopy, histology)	Possible regeneration but small sample sizes
Kuah et al. [70]	AT	3.9, 6.7	sIA injection of MSC + cell supernatant	Baseline, control group treated with cell supernatant and vehicle only	8 (3.9 × 10^6^ dose); 8 (6.7 × 10^6^ dose); 4 (control)	MRI (volume, MOAKS), ELISA (C2C, CTXII, HA, CTX-I)	0, 12 months	Chondroprotection within the low-dose group relative to high-dose and control group by MRI but not biomarkers

*M* bone marrow, *AT* adipose tissue, *WJ* Wharton’s jelly, *CB* cord blood, *sIA* single intra-articular injection, *HA* hyaluronic acid, *PRP* platelet-rich plasma, *MRI* magnetic resonance imaging, *MOAKS* MRI Osteoarthritis Knee Score, *MOCART* magnetic resonance observation cartilage repair tissue, *WORMS* whole-organ magnetic resonance imaging score, *ADC* apparent diffusion coefficient, *FA* fractional anisotropy, *KL* Kellgren-Lawrence classification of osteoarthritis, *JSW* joint space width, *HKA* hip-knee angle, *SafO* safranin O, *MSC* mesenchymal stromal cell, *MRA* Magnetic resonance arthrogram, *dGEMRIC* delayed gadolinium-enhanced MRI of cartilage, *COL1* collagen type I, *COL2* collagen type II, *COMP* cartilage oligomeric matrix protein, *C1-2C* C1-2C collagen type I and II cleavage, *C2C* collagen type II cleavage, *CTX-I* C‐telopeptide of type II collagen, *CTX-II* C‐telopeptide of type II collagen, *MMP-3* matrix metalloproteinase 3

The findings of studies involving the use of autologous MSCs have generally supported the cartilage reparative effects of MSCs, and these studies have included MSCs derived from either adipose tissue or bone marrow. For studies investigating autologous MSC(AT), doses range from 10 × 10^6^ to 100 × 10^6^ cells with single or multiple injections. A dose comparison study by Jo et al. using intra‐articular injections of autologous MSC(AT) reported a significantly increased cartilage volume and reduced size of cartilage defects in the medial femoral and tibial condyles of knee OA (KL grade 2–4) patients at 6 months after injection within their high-dose (100 × 10^6^ cells, *N* = 12 patients) but not lower-dose (10 × 10^6^ cells, *N* = 3) or moderate-dose (50 × 10^6^ cells, *N* = 3) groups [45]. The authors also showed through arthroscopic and histological assessment at baseline and 6 months the presence of regenerated cartilage and reduced cartilage defects [45]. However, in a follow-up Phase IIb RCT from the same investigators, more modest effects on cartilage were reported when comparing only the high-dose (100 × 10^6^ fresh cells) group vs. saline-treated controls (*N* = 12/group, KL grade 2–4); autologous MSC(AT) injections had no significant effect on the change in the size of cartilage defects at 6 months relative to baseline by MRI, while patients in the saline control group displayed significant exacerbations of cartilage defects, suggesting a chondroprotective but not a regenerative effect of the MSC(AT) [57]. Using the same dose of 100 × 10^6^ autologous MSC(AT) but with cryopreserved cells, Freitag et al. compared single versus two injections of autologous MSC(AT) (doses 6 months apart, KL grades 2–3) to conservative management control treatment (*N* = 10/group), reporting improved chondroprotection for the two-dose group relative to the single-dose and control groups at 12 months after injection as measured using the semiquantitative MRI Osteoarthritis Knee Score (MOAKS; a semiquantitative and subregional scoring system) method [58].

Regenerative effects of autologous MSC(AT) have also been reported at moderate doses. A dose comparison study investigating bilateral repeat injections of autologous MSC(AT) within both knee joints (10, 20 or 50 × 10^6^ cells/dose at 0, 3 and 48 weeks, KL grade 2–3; *N* = 4–5/group) showed increases in cartilage volume up to 72 weeks relative to baseline that favored the 50 × 10^6^ dose [126]. Interestingly, a subsequent decrease in cartilage volume was observed at 96 weeks (approx. 22 months) after injection [126], suggesting that regenerative effects may have been transient and emphasizing the importance of long-term follow-ups. A similar approach was used to evaluate bilateral repeat injections of autologous MSC(AT) (50 × 10^6^ cells/dose at 0 and 3 weeks; KL grade 2–3) relative to an HA control treatment (*N* = 26/group), reporting significantly increased cartilage volume at 48 weeks within the MSC(AT)-treated group, while cartilage degeneration was observed within the HA-treated group [61]. At the same dose level, Chen et al. showed that intra-articular injections (50 × 10^6^ cryopreserved cells) in knee OA (KL grade 2–3, *N* = 12) significantly improved articular cartilage pathology and several other MOAKS metrics at 48 weeks relative to baseline, including improvements in the overall MOAKS and bone marrow lesions, while no significant changes in the joint space height as measured by X-ray were detected [127]. Taken together, these data suggest that moderate to higher doses (≥50 × 10^6^ cells) of autologous MSC(AT) delivered through single or repeat injections can effect measurable cartilage repair changes, at least over shorter durations of 6–12 months. Longer studies are rare, but their findings suggest the absence of sustained cartilage reparative effects, which merits further investigation.

Dose ranges for studies investigating autologous MSC(M) are similar to those for studies on MSC(AT) but with a slightly wider range of 1 × 10^6^—100 × 10^6^ cells with single or repeat injections, as well as the use of combination strategies involving surgical interventions or delivery of HA or PRP. As detailed in the RCT section above, Lamo-Espinosa et al. compared single-dose intra-articular injection of MSC(M) combined with HA injection (10 × 10^6^ and 100 × 10^6^ fresh cell dosage groups) relative to HA injection alone (*N* = 10 patients/group) in knee OA patients (KL grade 2–4) and reported modest chondroprotective effects that favored a high dose, as evidenced by X-ray at 6 and 12 months showing a reduction in the knee articular interline within the HA group that was significant at 12 months, while no degenerative changes were observed in the MSC(M) treatment groups [60]. However, the same group administered three weekly doses of a combination product including autologous PRP with or without autologous MSC(M) (100 × 10^6^ fresh cells/dose, *N* = 30/group, KL grade 2–4) in a RCT and showed no significant effects on cartilage volume changes as evaluated by X-ray and the MRI WORMS protocol at 12 months after injection [60]. Other clinical trials conducted by this group in Spain have reported regenerative effects of single injections of autologous MSC(M) at moderate doses (40 × 10^6^ fresh cells) with significant improvements in cartilage quality for knee OA patients (KL grade 2–4) by T2 relaxation times (an MRI metric used to evaluate cartilage quality that is sensitive to water content and collagen fibril organization) and the poor cartilage index (PCI, an estimate of the percentage of T2 values above 50 ms) at 12 months relative to baseline [128–130]. Regenerative effects have also been reported by Al-Najar et al. using two intra-articular injections of autologous MSC(M) (at comparable doses of 30.5 × 10^6^ fresh cells/dose, 1 month apart, *N* = 13) in knee OA patients (KL grade 2–3), showing significant increases in cartilage thickness by T2 mapping at 12 but not 6 months after the first injection relative to baseline [131]. Work from our group has shown more modest effects of autologous MSC(M) on knee cartilage. In knee OA patients (KL grade 3–4) administered a single intra-articular injection of autologous MSC(M) (1 × 10^6^, 10 × 10^6^, 50 × 10^6^ cells, *N* = 4/group), we observed no changes in WORMS or T2 relaxation times at 6 and 12 months after injection relative to baseline for any cell dosage groups [119], including the high-dose group, which was comparable in magnitude to that in the Spanish studies. However, an analysis of cartilage breakdown byproducts by general estimating equations (GEEs) showed a significant decrease in the levels of serum C1‐2C in the 50 × 10^6^ cell dose group relative to the 1 × 10^6^ and 10 × 10^6^ cell dose groups [119]. Overall, the findings of clinical trials involving autologous MSC(M) indicate possible chondroprotective and regenerative effects and suggest that moderate-to-higher dose (≥40 × 10^6^ cells) single-injection autologous MSC(M), speaking to the basal fitness of autologous MSCs from OA patients or lower doses that are repeated or combined with other interventions, have measurable effects on cartilage. The evaluation of serum and urine biomarkers of cartilage breakdown products may be more sensitive than MRI evaluations of cartilage integrity, but a combinatorial approach is likely needed to understand all effects.

Using allogeneic MSCs, mixed effects of intra-articular injections on OA cartilage have been reported, and this may be related to greater use of cryopreserved cells, the use of other MSC tissue sources that have been less studied in the context of OA clinical trials, the use of combined interventions and an overall fewer number of allogeneic trials, all of which complicate interpretations. In a RCT, Soltani et al. showed regenerative effects of allogeneic placenta-derived MSCs (50–60 × 10^6^ fresh cells), as evidenced by significantly increased cartilage thickness at 24 weeks relative to baseline in knee OA patients (KL grade 2–4), while no changes were observed with saline vehicle control (*N* = 10/group) [67]. A RCT by Vega et al. comparing a single-dose intra-articular injection of allogeneic MSC(M) (40 × 10^6^ fresh cells) to HA injection (*N* = 15/group) in patients with knee OA (KL grade 2–4) also reported regenerative effects on cartilage with significant improvements in cartilage quality by the PCI in the MSC(M) group at 12 months relative to baseline that was not apparent in the HA group [68]. Another trial in which patients with knee OA (KL grade 2–3) received bilateral repeat doses of allogeneic MSC(AT) (10 × 10^6^, 20 × 10^6^, or 50 × 10^6^ cryopreserved cells/dose in both knees at 0 and 3 weeks, *N* = 7–8/group) also showed improvements in cartilage volume and integrity by MRI across dosage groups at 48 weeks relative to baseline [132, 133]. Using multiparametric MRI measurements (including WORMS, T2, and other metrics), the authors reported that T_1rho_ measurements (estimating glycosaminoglycan (GAG) and proteoglycan content) were the most sensitive for detecting differences between dose groups, with the high-dose group showing significantly greater increases in T_1rho_ values [132]. In contrast to these studies, a RCT by Matas et al. comparing single and two doses of MSC(WJ) (20 × 10^6^ fresh cells/dose, with a second dose at 6 months for the two-dose group) to HA injection (*N* = 8–10/group) in knee OA patients (KL grade 1–3) showed no detectable changes in MRI WORMS measured at 6 and 12 months after injection [69].

Allogeneic MSCs have also been investigated in combination with other injectables. Using cryopreserved allogeneic MSC(AT) and cell culture supernatant containing MSC-secreted factors and EVs, Kuah et al. reported evidence of chondroprotection in a RCT with knee OA patients (KL grade 1–3); patients receiving the low-dose cell product (3.9 × 10^6^ cryopreserved cells, *N* = 8) showed no reduction in cartilage volume, while significant reductions were observed in both the high-dose (6.7 × 10^6^ cryopreserved cells, *N* = 8) and control (cell culture media and cryopreserved cells, *N* = 4) groups at 12 months relative to baseline [70]. Notably, the authors reported no changes in serum or urine levels of cartilage catabolic biomarkers (C2C, CTX-II, HA, and CTX-I) [70], which contrasts with findings from our previous study showing reductions in catabolic biomarkers but no detectable cartilage volume changes by MRI [119]. A RCT by Gupta et al. in which knee OA patients (KL grade 2–3) were administered cryopreserved allogeneic MSC(M) from pooled donors (25 × 10^6^, 50 × 10^6^, 75 × 10^6^, or 150 × 10^6^ cells/dose in separate cohorts, *N* = 10/cohort) or vehicle (*N* = 5/cohort) followed by HA injection demonstrated no effect of the MSC injections on cartilage volumes as assessed by X-ray and MRI WORMS scores relative to baseline or control at 6 and 12 months follow-up [65]. Using an in situ tissue engineering strategy, Park et al. implanted a composite consisting of a HA hydrogel with allogeneic MSC(CB) (0.5 × 10^7^ cells/mL and 500 µl/cm^2^ of the defect area, *N* = 6) into cartilage defects for patients with knee OA (KL grade 3) and ICRS grade 4 cartilage lesions [134]. Arthroscopic evaluation of the defects at 12 weeks postimplantation revealed maturing cartilage, and ICRS grades were reduced in 4 out of 6 patients. A subset of two consenting patients at the 1-year follow-up showed hyaline-like cartilage that integrated with the surrounding tissue, along with positive staining with the proteoglycan-binding dye safranin O and cartilage-specific collagen II by biopsy. MRI measurements at the 3-year follow-up (*N* = 5) also showed increased GAG content at the defect sites relative to baseline using delayed gadolinium‐enhanced MRI of cartilage (dGEMRIC). Notably, the authors reported significant improvements in VAS pain scores and IKDC scores at 6 months relative to baseline that were maintained through 7 years of follow-up in an analysis of a subset of patients without additional knee surgery or replacements [134]. This tissue-engineered product has been commercialized in S. Korea as CARTISTEM™; it is currently in clinical evaluations in the USA (NCT01733186) and Japan.

Taken together, recent data support the regenerative and/or chondroprotective effects of intra-articular MSC injections, especially when injected at moderate to higher doses (≥40 × 10^6^ cells), in repeat doses or when combined with surgical interventions. However, differences in the baseline clinical phenotype of patients, MSC tissue sources, the use of fresh versus cryopreserved cells, and the use of combination strategies involving coinjections with HA or PRP and surgical interventions confound these analyses. Additional high-quality evidence is required with higher powered and well-controlled clinical trials coupled with longer durations of follow-up for evaluating cartilage changes by MRI. Measurements of biomarkers for cartilage degradation in synovial fluid, urine, serum and plasma along with patient clinical phenotyping and endotyping will also provide useful insights into this potential mechanism of action of MSCs in effecting cartilage regeneration and repair in knee OA. Importantly, the reported clinical chondroprotective and regenerative effects of MSCs are observational and may be achieved through several mechanisms. Donor MSCs may directly regulate the endogenous chondrocyte phenotype by modulating their ECM production and promoting cell survival and autophagy via growth factors, microRNAs (miRs), EVs and organelle transfer [135–138]. MSCs may also recruit endogenous progenitors [139] and regulate their differentiation into chondrocytes [140]. Alternatively, MSCs can modulate the joint microenvironment to indirectly promote cartilage repair through interactions with immune cells and other joint tissues, as discussed in the previous subsections. Thus, further study is needed on the multimodal mechanisms of action of MSCs, but these mechanisms are likely to include both direct and indirect effects on cartilage, as previously reviewed [74, 141].

### Other potential mechanisms

In addition to having immunomodulatory and chondroprotective effects in OA, MSCs may act through a variety of other mechanisms that target multiple tissues within the joint. These mechanisms include antifibrotic and angiogenic effects, which are interrelated with immunomodulation. In particular, the pro-angiogenic mechanisms of MSCs have been widely documented and are of clinical relevance to a variety of conditions, such as critical limb ischemia [142]. The potential pro-angiogenic effects of MSCs are understudied in OA, but angiogenesis is generally associated with increased inflammation, synovitis, and pain [143]. However, our previous clinical study in knee OA demonstrated a significant increase in levels of the pro-angiogenic factor VEGF in the synovial fluid of OA patients at 3 months relative to baseline after autologous MSC(M) injections, and this was observed in parallel to significant improvements in patient pain and other PROMs, as well as reduced joint inflammation [119]. VEGF has been shown to be associated with catabolic processes in synovial cells and chondrocytes in a mouse surgically induced OA model [144]; however, other pro-angiogenic factors, such as FGF2, which can also be produced by MSCs, may have pleiotropic functions in OA, with FGF2 having dichotomous effects on articular chondrocytes dependent on relative chondrocyte expression of FGFR1 and FGFR3 receptors [145]. Moreover, platelet-rich plasma (PRP), an often used analgesic for joint pain and inflammation, is known to be rich in pro-angiogenic factors, including PDGF isoforms, TGF- a/TGF- b, VEGF, EGF, FGF-a/b, connective tissue growth factor (CTGF), IGF-1, hepatocyte growth factor (HGF), keratinocyte growth factor (KGF) and Ang-1, and yet symptom and pain-relieving effects of PRP injections have been reported in RCTs and in several meta-analyses in knee OA patients [146–148].

There is a complex interplay between inflammatory and angiogenic processes, with immune cells playing a critical role in the temporal regulation of vessel remodeling through both pro- and anti-angiogenic mechanisms [149]. Previous work by Boregowda et al. indicated that the in vitro immunomodulatory and pro-angiogenic properties of human MSC(M) may be inversely correlated and mediated by levels of the transcription factor TWIST1 [150]. The stimulation of MSC(M) with proinflammatory factors increased the immunosuppressive functions of MSC(M) and reduced the levels of TWIST1, while the stimulation of MSC(M) with pro-angiogenic factors had the opposite effect [150]. In support of this, we have separately shown that lower levels of several angiogenic factors inversely correlated with the immunomodulatory fitness of human MSC(AT), as evaluated by in vitro monocyte/macrophage polarization toward inflammation-resolving phenotypes [91]. Thus, it is possible that the immunomodulatory properties of MSCs may predominate over the angiogenic properties, and this could be mediated by proinflammatory cues within the joint microenvironment in knee OA; however, this is speculative, and there is likely a complex interplay between inflammatory and angiogenic processes mediated by MSCs for which further study in an OA context is needed.

Antifibrotic effects of MSC therapies have been documented in several clinical indications for which fibrosis is a disease hallmark, including for idiopathic pulmonary fibrosis [151] and ischemic cardiomyopathy [152]; thus, antifibrotic functions represent a possible mechanism of action for MSCs in OA. Fibrotic tissue in the synovium and cartilage is marked by synovial hyperplasia and fibrocartilage formation, respectively, which modulate the disease tissue microenvironment and cellular phenotypes within the OA joint [153]. Importantly, a complex interplay exists among tissue fibrosis, inflammation, and angiogenesis, and MSCs can modulate immune cells and augment neovascularization to remodel fibrotic tissue, as we discussed in a previous review [154]. While reduced synovial hyperplasia has been shown in preclinical OA studies evaluating MSC treatment [105, 155], more research is needed to understand whether MSCs may exert direct antifibrotic effects on OA or whether this is achieved indirectly through MSC-mediated immunomodulation or angiogenesis.

In addition to the immunomodulatory, angiogenic, and antifibrotic properties of MSCs that may impact multiple tissues within the joint, MSCs may have tissue-specific effects on the subchondral bone, menisci, and ligaments. MRI data on these tissues from clinical studies involving MSCs in OA are sparse and limited by the sensitivity of the technique. However, Chen et al. have shown that autologous MSC(AT) injection significantly reduced the MOAKS for bone marrow lesions and cysts, meniscus pathology, and periarticular features at 48 weeks relative to baseline, while no significant differences were observed for osteophyte, synovitis, ligament tears or tendon abnormality scores [127]. In terms of subchondral bone, MSCs can interact with osteoclasts in OA through direct cell‒cell contact and indirectly through secreted factors and EVs to inhibit osteoclastogenesis and subchondral bone resorption, as recently reviewed [156]. Osteoblasts are also known to modulate cartilage metabolism and subchondral bone remodeling in OA, and a previous in vitro study demonstrated that EVs derived from human MSC(AT) reduced OA osteoblast senescence and the release of inflammatory mediators such as IL-6 [157].

The menisci and ligaments of the joint play important roles in mechanical support, and damage to these tissues can precede radiographic OA; however, their roles in OA progression and pathophysiology are poorly understood [158]. A recent study by Ramos-Mucci et al. demonstrated that the ligaments and menisci undergo structural changes, including increased mineralization and chondrogenesis, in both a spontaneous and surgically induced OA model [159]. MSCs are being investigated for meniscal and ligament repair due to their chondroprotective and immunomodulatory properties [160, 161], and thus, the joint menisci and ligaments represent possible additional target tissues for MSC mechanisms of action in OA that merit further study.

### Influence of MSC immunomodulatory basal fitness on their mechanisms of action

Several factors can influence the fitness and overall phenotype of MSCs that may in turn influence their mechanisms of action, as discussed here with emphasis on effects mediating immunomodulatory fitness. Donor heterogeneity represents a major variable and includes a multitude of factors, such as donor health status, age, BMI and sex, that can influence the in vitro clonogenic potential, paracrine functions, and proclivity toward senescence of MSCs [162–165]. We have been investigating the interactions between donor heterogeneity and the optimization of culture variables to culture-expand MSC(M) while maintaining their immunomodulatory functionality and have shown that donor heterogeneity is an important variable for a subset of donors; surrogate gene readouts are also being developed to prospectively identify these donors (Kolade et al., manuscript in preparation). Tissue procurement, isolation and manufacturing strategies for MSCs are also factors that modulate MSC behavior [166, 167]. MSC donor fitness is typically minimally investigated by evaluating cell viability, proliferation and trilineage differentiation; these parameters are often separate from the actual clinical mechanisms of action in OA and thus provide limited insights into the true fitness of MSCs prior to intra-articular injection. More rigorous assessments, as we have recently proposed [91], are needed to better understand and evaluate basal MSC donor fitness [168]. The level of expansion for MSCs should also be carefully monitored, as transcriptome drift has been reported to precede changes in growth kinetics and other hallmarks of senescence for human MSC(WJ) [169].

In support of the immunomodulatory mechanism of action of MSCs in OA, we and others have investigated the relationships between the baseline immunomodulatory fitness of donor MSCs in vitro and their clinical efficacy. In our clinical trial, we measured the levels of IFN‐γ-induced expression of immunomodulatory genes (prostaglandin-endoperoxide synthase 2, *PTGS2*; cluster of differentiation 274*, CD274*; indoleamine 2,3-dioxygenase 1 *IDO1*; interleukin-10, *IL10*; hepatocyte growth factor, *HGF*; and transforming growth factor beta 1, *TGFB1*) and TNF-α-induced TSG6 protein expression by MSC(M) in vitro and found correlations with changes in WOMAC and KOOS scores [69]. These data suggest that MSC(M) with higher baseline immunomodulatory fitness levels in vitro afforded improved patient outcomes, thus supporting immunomodulatory functionality as a clinically relevant mechanism of action for MSC(M). In support of these findings, Chen et al. showed that the levels of intracellular IDO protein expression in IFN‐γ-stimulated MSC(AT) in vitro were significantly correlated with in vitro suppression of T-cell proliferation as well as improvements in KOOS sports, VAS and IKDC scores in OA patients receiving intra-articular injections of MSC(AT) [127]. Notably, while the findings of both studies [119, 127] support an immunomodulatory mechanism of action of MSCs in OA, neither study found changes in synovitis scores by MRI [119, 127], pointing to the relative insensitivity of MRI in detecting changes in synovial inflammation, at least over shorter time intervals [127]. Matas et al. have also reported the measurement of secreted thrombospondin-2 to select MSC(WJ) donors in their RCT for OA, citing the role of thrombospondin-2 in regulating chondrogenic differentiation [69]. Interestingly, thrombospondin-2 has also been shown to exert anti-inflammatory effects in rheumatoid arthritis models [170], suggesting that the MSC(WJ) used in Matas et al. may also have exhibited augmented immunomodulatory fitness, at least in vitro.

Delivery strategies are also likely to influence mechanisms of action, and the administration of freshly thawed cryopreserved MSCs can influence their cell viability and apoptotic status [171] and diminish their immunosuppressive capacity [172]. Cryopreservation also influences cell membrane integrity, which in turn facilitates complement binding and subsequent innate effector cell recognition [173]. Evidence from Antebi et al. demonstrated that 24-h in vitro culture post-thaw provided sufficient recovery time for human MSC(M) to regain the expression of angiogenic and anti-inflammatory genes as well as in vitro T-cell immunosuppression [174]. Interestingly, the application of MSC(CB) commercialized by MEDIPOST as CARTISTEM® for the treatment of degenerative arthritis includes a culture-rescue period in the final passage prior to surgical implantation to treat cartilage defects [175]. Nonetheless, positive effects on PROMs and cartilage repair have been reported using both cryopreserved and fresh or culture-rescued cells, although these have not been directly compared in OA clinical trials. While fresh low-passage MSCs may be expected to have higher baseline therapeutic fitness, there are conflicting preclinical data, and the interplay between the combinatorial effects of fresh/frozen status, freeze/thaw methods, and other manufacturing or donor-specific variables collectively influences disease-specific mechanisms of action and overall treatment efficacy, as has been recently reviewed [173].

The tissue sources for MSCs represent an important variable that could also influence MSC mechanisms of action. To date, bone marrow, adipose tissue and umbilical tissues represent the most commonly used MSC sources in OA clinical trials. An elegant study by Ménard et al. investigated matched human MSC(AT) and MSC(M) from 14 donors and demonstrated distinct transcriptional profiles that were imprinted by the tissue of origin in each MSC population, with different sets of immunomodulatory genes and chemokines upregulated in MSC(AT) and MSC(M). MSC(AT) displayed reduced HLADR expression, as well as augmented neutrophil recruitment and suppression of T-cell proliferation in vitro, whereas MSC(M) were better able to suppress natural killer (NK) cell proliferation in experiments with paired donors [176]. Binch et al. also investigated the responses of donor-matched MSC(M) and MSC(AT) to proinflammatory cytokine stimulation and hypoxia, reporting that MSC(AT) upregulated angiogenic and neurotrophic factors under hypoxic and proinflammatory conditions relative to MSC(M) [177]. The findings from these studies using donor-matched MSCs from multiple tissue sources offer important insights into the effects of the tissue of origin on the functional properties of MSCs. While cord blood and Wharton’s jelly also represent common MSC sources, the fetal origin of MSC(CB) and MSC(WJ) prevents donor-matched comparisons to MSC(M) or MSC(AT). Nonetheless, several studies have compared the gene expression profiles and immunomodulatory properties of MSC(AT), MSC(M), MSC(CB), and MSC(WJ) from different donor populations [178, 179], but the optimal tissue source in the context of OA remains unclear and may be dependent on individual donor and manufacturing parameters. Furthermore, there are limited clinical data in OA to draw reliable conclusions on the most suitable tissue source for MSC procurement. Indeed, meta-analyses comparing MSC(M) and MSC(AT) have reached conflicting conclusions, with the findings of one study supporting the improved clinical efficacy of MSC(M) [78], while the findings of other studies support MSC(AT) [81, 180, 181]. Thus, additional research is needed to investigate tissue-dependent effects on MSC treatment efficacy.

The choice of autologous versus allogeneic MSCs is another factor that may impact mechanisms of action. An advantage of allogeneic MSC sources is that they offer the ability to select healthy donors and screen for MSCs with high fitness levels, as we have postulated [91]; we also noted significant donor-driven heterogeneity that correlated with clinical effectiveness [119] (Robb et al., manuscript in preparation). MSCs from allogeneic sources can be expanded in large quantities to treat multiple patients and used off-the-shelf, reducing the cost of manufacturing in an industry driven by cost margins [182]. However, immune recognition is a consideration for the use of allogeneic MSCs for which further study in OA is needed, especially for repeat injections and at higher cell doses. Equine studies have documented alloantibody production in serum after intra-articular injection of allogeneic MSCs, including in a chemically induced OA model where lower antibody responses were observed in half-matched haplotype donors and in proinflammatory cytokine-primed MSC(M) relative to mismatched naïve MSC(M) after a second injection [183], and in healthy joints where cytotoxic antibodies were observed in response to allogeneic but not autologous synovial membrane-derived MSCs [184]. A RCT by Wang et al. reported alloantibody detection in anterior cruciate ligament (ACL) injury patients who underwent ACL reconstruction followed by intra-articular injection of the allogeneic MSC(M) Mesoblast product (single injection, 75 × 10^6^ cryopreserved cells) combined with HA injection. However, significant improvements in KOOS and 36-item Short Form Survey (SF-36) scores and reduced joint space narrowing were observed in the MSC(M)+HA group relative to the HA alone group, suggesting that allorecognition did not preclude treatment efficacy in this study [185]. To our knowledge, clinical data on alloantibody production against MSCs have not been reported in OA; indeed, improved efficacy of repeat versus single injection of allogeneic MSC(WJ) has been reported [69]. The study by Gupta et al. using a single injection of allogeneic MSC(M) from pooled donors (Stempeucel®) also showed greater albeit nonsignificant improvements in PROMs at lower doses relative to moderate and high doses [65]. While not tracked in either of these studies, alloantibodies may have been produced; however, further research is needed to understand whether these may have adverse effects on patient outcomes or side effects for consideration of repeat injection, dosing and use of pooled allogeneic versus single MSC donors. [183], and in healthy joints, where cytotoxic antibodies were observed in response to allogeneic but not autologous synovial membrane-derived MSCs [184].

In contrast, autologous sourcing would circumvent alloantibody production, but donor site morbidity and variable basal MSC therapeutic fitness, which can be impacted by donor factors including age, sex, BMI, physical activity and health status, must be factored in with its use. For example, MSCs obtained from older donors may be more prone to senescence, which may limit the cell numbers required for specific dosing, skew their secretory phenotype and diminish their functional immunomodulatory properties [186]. Thus, autologous MSC treatment efficacy could be limited to individuals for whom adequate doses of potent autologous MSC products can be obtained. Other strategies to enhance “nonfit” autologous MSCs may also be considered, including in vitro priming with exogenous cytokines [187], hormones, growth factors, or senolytic agents, as well as modulating matrix mechanics, oxygen tension, and the use of 3D culture formats [167, 188]. Genetic engineering approaches may also be considered to enhance MSC potency, including revitalizing the paracrine functionality of culture-expanded MSCs through transduction with a cocktail of transcription factors, as reported by Nakahara et al. [189].

### Summary of current knowledge on MSC mechanisms of action in OA

There is limited clinical evidence on MSC mechanisms of action in OA. Recent clinical data have supported immunomodulation as a relevant mechanism of action for MSCs in OA [119–121, 127], but more research is needed to understand the systemic and local effects of intra-articular MSC injections on cellular and humoral immune responses. Clinical trials have shown that MSCs may exert cartilage reparative and/or chondroprotective effects on OA, as evidenced by imaging, second-look arthroscopy, histology, and biomarker analyses; however, additional higher-powered and controlled studies are needed to confirm these findings. While these results are promising and suggest disease-modifying effects, they provide little insight into clinical mechanisms of action given that MSCs may achieve these effects directly through the release of chondroprotective or growth factors, indirectly through the modulation of the joint microenvironment, or through some combination of the two. Similarly, investigations on antifibrotic and angiogenic mechanisms combined with greater attention to all joint tissues, including the synovium, infrapatellar fat pad, subchondral bone, menisci, and ligaments, are needed. Importantly, MSC tissue sources, donor factors, manufacturing parameters, and delivery strategies should be carefully considered, as these may impact the mechanisms of action and overall therapeutic efficacy. Altogether, an improved understanding of MSC mechanisms of action in OA could accelerate efficacious clinical translation, especially when coupled with knowledge of OA patient clinical phenotypes and endotypes to select patients for MSC therapies, as discussed in the subsequent section.

## Roadmap for successful MSC OA trials

### Selecting the right OA patient endotype for MSC therapies

OA is a heterogeneous disease, and at least six clinical phenotype subsets have been identified [12], including mechanical, chronic pain, metabolic, inflammatory, bone and cartilage phenotypes and minimal joint disease, representing different combinations of symptom manifestations [13]. Additionally, patients need to be classified into more complex and overlapping molecular endotypes (which include existing soluble biomarkers (reviewed in [190]). Emerging transcriptomic, lipidomic, and miR profile datasets (reviewed in [190]) will provide insights into molecular mechanisms that drive the multiple clinical phenotypes, and this represents an active area of research in better defining OA pathogenesis [13].

Categorizing OA patients with the inflammatory phenotype (based on synovial inflammation) may be most relevant to MSC therapies with their proposed anti-inflammatory mechanisms of action. Indeed, the current most effective therapeutic treatments for OA symptoms and pain are nonsteroidal anti-inflammatory drugs (NSAIDs) and intra-articular corticosteroid injections that work through anti-inflammatory effects on synovial inflammation with large effect sizes [191]. Synovial inflammation is associated with joint failure [192] and is characterized by macrophage infiltration and activation; our group has shown an inverse correlation between PROMs and the frequency of specific synovial fluid MΦ subsets in knee OA patients [97]. Given the immunomodulatory functionality of MSCs, particularly their important effects on host MΦs [99], targeting patients with molecular endotypes of inflammation for MSC treatments may be the most expedient first step in stratifying patients clinically. However, identifying this subset of patients is not easy and is postulated to be one of the reasons why clinical trials of therapies targeting inflammation, including anti-IL1 and anti-TNF therapies, have failed [193]. Indeed, subset analyses in some of these studies show significant effects, for example, of etanercept (TNF blocker) in symptomatic hand OA patients (identified by swelling/erythema and positive power Doppler by ultrasound (US) imaging) who showed significant improvements in pain and structural damage [194] when the overall cohort did not.

Part of the conundrum is that it is not clear that an inflammatory phenotype is a true OA clinical phenotype [191] or is just part of the continuum of OA pathogenesis and severity, happening on fluctuating temporal scales, dependent on obesity, physical activity and other personalized metrics [115]. Indeed, tools for measuring clinical inflammation (which may not fully reflect molecular endotypes) are varied with different sensitivities, including contrast- and noncontrast-enhanced MRI measurements [195], US measurements of synovial effusion [196], and serum biomarkers such as the degradation of C-reactive protein (CRP), connective tissue type 1 collagen turnover (C1M), and matrix metalloproteinase 3 (MMP3) [197]. Serum and local synovial fluid biomarkers may be more sensitive and reflective of molecular endotypes. However, all these measurements may be confounded by changes in body mass index and physical activity [115].

US imaging has been postulated to be one such measurement modality for identifying patients with increased inflammation [13] and has been shown to correlate with histopathological findings [198]. Indeed, in our MSC(M) knee OA trial, we used US imaging to identify patients with synovial fluid accumulation at the time of enrollment as an inclusion criterion that facilitated baseline measurements of synovial fluid immune cell subsets and proinflammatory mediators [119]. Our preliminary assessment showed interesting correlations of baseline levels of biomarkers for inflammation and cartilage degradation with patient pain and function responses at follow-up timepoints up to 24 months after single injections of autologous MSC(M) intra-articular injections (Robb et al., manuscript in preparation).

Ultimately, the use of combinatorial tools, including US and MRI, serum biomarkers, and synovial fluid levels of immune cell subsets and proinflammatory mediators, will help identify true inflammatory patient endotypes (vs. clinical phenotypes) that might be most receptive to MSC treatments based on their proposed anti-inflammatory and immunomodulatory effects, particularly on host MΦs. Flow cytometry evaluations would thus be valuable to profile cellular responses within local joint biopsy samples, including immune cell profiling of synovial fluid as we have previously shown [97, 119], as well as mass cytometry approaches as employed by Yager et al. [199] Importantly, we [97] and others [200, 201] have shown divergent profiles of local vs. circulating immune cells in OA, emphasizing the need to sample joint-specific tissues and fluids for more accurate representations of disease endotypes. Indeed, profiling patient-specific immune cells to understand host immune responses and their interactions with exogenously administered MSCs will be key to understanding and selecting appropriate patient endotypes that are responsive to MSC therapies and thus to designing successful clinical trials in OA patients [202].

The identification of circulating miR biomarkers [203] using unbiased sequencing and other omics-based approaches (genome-wide association studies [204], proteomics [205], metabolomics [206]) may provide deeper insights into patient endotype. The use of AI algorithms and bioinformatics to cohesively knit large omics datasets [207] to systematically classify patient endotypes can further advance the field. Organ-on-chip technology, which can incorporate patient-specific blood, synovial fluid, urine and/or tissue biopsy samples, offers the dual potential of providing patient baseline disease endotype information and surrogate in vitro readouts of the efficacy of various drugs or test therapeutics [208].

### Designing the right MSC therapeutic product for OA

As discussed above, the immunomodulatory basal fitness of MSCs, which can be modulated by age, sex, BMI, comorbidities, manufacturing parameters and cryopreservation and can be quantified by a range of quantitative CQAs [91], is an important parameter in selecting an appropriately fit MSC therapeutic product [202]. Similar to their utility for patient endotyping, omics-based approaches may be useful to profile donor MSCs that achieve efficacious outcomes in OA clinical trials to establish correlative CQAs that correspond to beneficial patient responses and are relevant to disease-specific mechanisms of action in OA. Indeed, using MSC(M) patient samples from our previous OA clinical trial with autologous MSC(M), we performed miR sequencing and identified miR candidates that correlated with PROM improvements (Robb et al., manuscript in preparation). These miRs are currently under investigation and will provide useful insights into MSC mechanisms of action in OA and/or be used as CQAs to define MSC immunomodulatory basal fitness (Robb et al., manuscript in preparation).

Functional high-content in vitro potency assays that are relevant to the mechanisms of action may also be useful for characterizing MSCs to better understand the influence of basal fitness levels on treatment efficacy [209]. MSC(WJ) donor selection based on in vitro proliferation, trilineage differentiation and the secretion of thrombospondin-2 (a regulator of chondrogenic differentiation) was recently applied by Matas et al. in selecting basally fit MSC(WJ) for treatment groups in their RCT, from which they reported efficacious outcomes of MSC treatment [69].

As discussed above, dosing, carrier choice and selection are also not fully understood, and in many cases medium to high (≥40 × 10^6^) doses of MSC(M) or MSC(AT) seem to provide more beneficial analgesic and cartilage structural improvements than lower doses. Including the combination of MSCs and HA or PRP or MSCs with surgical approaches has shown mixed effects on MSC-mediated chondroprotection or cartilage repair, depending on the study. To this end, cell tracking studies could provide useful insights. We have done this using iron nanoparticles approved for iron-deficiency anemia in the USA and Canada [111] to label mouse syngeneic MSC(M) and track them in a mouse surgical model of OA. Translating this to clinical trials and using MRI contrast agents such as Ferraheme® would advance our understanding of cell distribution, retention, and mechanisms of action in the joints of immunocompetent OA patients and help us better understand the MSC dosing needed for successful anti-inflammatory, analgesic and structural outcomes.

Limited short-term clinical evidence supports repeat dosing of autologous and allogeneic MSCs, albeit without a full understanding of the potential implications of alloreactions to repeat allogeneic MSC dosing. Clinical trials using allogeneic MSCs should incorporate measurements of humoral and cellular responses to better discern the correlation, if any, between alloreactions and reduced clinical efficacy. Importantly, data on longer-term structural changes as well as symptom and disease modification are not sufficient to recommend repeat injections, but they merit investigation in future clinical trials.

Ultimately, there are conflicting data regarding dosing from the RCTs and non-RCTs, and this seems to be driven in part by the baseline patient characteristics or clinical phenotyping; from our analyses, it appears that often the patients receiving lower doses of MSC products seem to have lower baseline pain/symptom scores, which confounds the analysis on dosing. Of the 15 RCTs, we noted differences in dose ranges tested for autologous (4.11–100 × 10^6^) vs. allogeneic (3.9–150 × 10^6^) MSCs, but we noted that this included different patient baseline characteristics; different delivery of PRP, HA, or corticosteroids; and different implementation of surgical interventions, all of which make it difficult to draw reliable conclusions regarding dosing. Moderate to higher dosing (≥40 × 10^6^ MSCs) or repeat dosing does seem to result in more cartilage structural changes [45, 68, 126, 129, 131], but larger sample sizes, longer-term patient follow-up, and baseline patient phenotyping and endotyping are needed.

Next-generation MSCs, with improved immunomodulatory basal fitness and designed to be fit-for-purpose (for OA treatment), are also being evaluated preclinically and clinically. Small-diameter MSC(CB) grown in hypoxic conditions in calcium-rich medium with improved basal fitness, including the ability to polarize MΦs to proresolving subtypes [210], are currently being clinically tested in mid-stage OA patients in S. Korea [211]. Other non-genetic physical modification methods to enhance MSC basal fitness include transient 3D culturing to improve basal immunomodulatory fitness [91], pulsed electromagnetic field therapy to increase basal MSC chondrogenesis via TGF-β signaling [212, 213], and low-level laser therapy-primed MSCs (rev. in [214]) to improve the proliferative index.

Induced-pluripotent stem cell (iPSC)-derived MSCs (iMSCs) represent another next generation MSC product currently under clinical evaluation for the treatment of tibiofemoral knee OA in a Phase III double-blinded, randomized, controlled trial [215]. These allogeneic iMSCs will be repeatedly injected (3x) into patients randomized to the treatment arm at weeks 1, 3 and 52 at 25 × 10^6^ cells. Importantly, the iMSCs are derived from iPSCs from a single donor, eliminating the heterogeneity challenge of using MSCs sourced from multiple donors to manufacture multiple batches needed to generate sufficient cell numbers for clinical evaluations. The safety and efficacy of injecting iMSCs will be evaluated over 24 months using pain, function and cartilage thickness measurement instruments [215]. iPSCs can be expanded as needed to generate multiple differentiated batches of relatively homogenous MSCs, ostensibly without fatiguing the MSCs or having them become senescent, as has been tested in clinical trials for GVHD [216]. However, iPSCs are not without risks, including the tumorigenicity, allorejection and heterogeneity of mixed populations containing differentiated and partially differentiated cells. The elimination of residual undifferentiated cells through various technologies [217, 218], including the incorporation of inducible suicide genes, will be a salient safety feature [219] to enable the commercialization of iPSC-differentiated cells. Different protocols to generate iPSC-derived chondrocytes, including IPSC-MSC-chondrocytes, are also being evaluated for cell-replacement therapies in OA (rev. in [220]). If iMSC OA trials are successful, this could also pave the way for the use of genetically modified iMSCs [221].

MSC-derived EVs with cargo consisting of proteins, mitochondria, miRs, ions, and lipids are also being evaluated preclinically [222, 223] for the treatment of OA. The donor heterogeneity, fitness, dosing, and mechanism of action challenges that surround MSCs will also need to be addressed for MSC-derived EVs for OA. MSC EVs for OA are currently being evaluated for safety and feasibility in Phase I/II trials (NCT05060107; NCT04314661; clinicaltrials.gov). Engineered EVs that contain cargoes of high levels of one or more key factors or exogenous therapeutic molecules are also being evaluated in various indications and will likely be of interest for OA therapeutics [224–226]. Identifying the key factors while maintaining a cocktail of other factors will require an in-depth understanding of OA etiopathogenesis to circumvent the failure of single tested therapeutics [224–226].

In summary, there are several technologies to aid in the selection or engineering of basally fit autologous or allogeneic MSC therapeutic products or engineering MSC-derived EVs that can be specifically curated for OA therapeutics. Ultimately, MSCs with multimodal anti-inflammatory, immunomodulatory, chondroprotective and antifibrotic functionalities will likely be of greatest benefit. Dosing remains an open question, although meta-analysis results seem to suggest that cartilage structural changes are feasible with >40–50 × 10^6^ MSCs [81, 89]. Dosing needs to be understood in the context of baseline patient characteristics, both endotype and phenotype, and the interactive effects of MSC dose, MSC fitness, and baseline disease burden.

### Considerations for successful clinical trial design for MSC products for OA

**Fig. 3:**
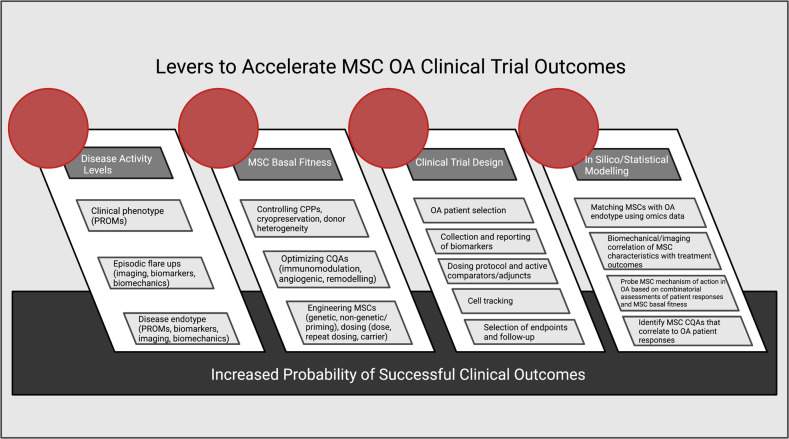
Levers for enabling successful mesenchymal stromal cell trials in osteoarthritis - Four aspects that need to be addressed to enable successful patient outcomes. PROMs patient-reported outcome measures, CPPs critical process parameters, CQAs critical quality attributes, MSCs mesenchymal stromal cells, OA osteoarthritis

Clinical trials investigating MSC treatments in OA should incorporate additional readouts on putative mechanisms of action to facilitate future regulatory approvals of MSC products in OA and to tailor next-generation MSC therapies that have specific therapeutic properties and CQAs that are tuned for relevance in OA (Fig. 3). MSC mechanisms of action in OA should be investigated from at least two different perspectives: (i) detailed analyses of patient OA molecular endotype, clinical phenotype and responses to MSC treatment and (ii) detailed basal characterizations of therapeutically fit MSCs. Analyses of patient factors could involve liquid and, if permitted by ethics boards, tissue biopsy specimens collected locally from the joint and systemically at baseline and subsequently post-MSC injection. Tissue specimens could include synovial fluid, blood, urine and cartilage or synovium collected through arthroscopic debridement. Samples collected at baseline may provide insights into both the clinical phenotype and the molecular endotype based on diverse selection of biomarkers (using proteomics, metabolomics, transcriptomics, miRs, etc.), imaging modalities and PROMs and can be used to inform future patient stratification to receive precision-matched MSC therapies. Samples collected at follow-up timepoints can be used to help determine the effects of MSC injections on inflammatory, catabolic, anabolic, and fibrotic biomarkers, as we have shown previously [119].

The episodic nature of OA, termed “flare-ups”, is poorly understood etiologically [227]; these flare-ups can be of varying durations and intensities and involve not only the exacerbation of pain, stiffness, and swelling of the joint but also the psychological and other consequential aspects of the flare-ups [228]. Appropriate timing of delivery for MSC therapies to coincide with flare-ups might result in both short-term symptom- and disease-modifying effects and longer-term disease-modifying sequalae, as OA is being better understood as an “acute-on-chronic" disease [227]. However, current instruments may be insufficient to investigate the temporal kinetics of “flare-ups”. Clinical trials can be designed with combinatorial measurement tools, including patient-reported data, biomarker and biomechanic evaluations [229–231], to capture the extent and frequency of “flare-ups”. Correlation between the occurrence of “flare-ups” and patient responses to MSC treatments in the short and longer term will inform future patient stratification strategies and timing of MSC treatments relative to OA “flare-ups”.

There are existing guidelines for clinical trial design considerations, including the selection of OA patient populations, as put out by the European Medicines Agency (EMA) [232]. This guideline spells out the following multiple design requirements: (i) the need for including patients with baseline symptomatic and structural changes; (ii) documenting patient characteristics (clinical phenotype), including the extent of disease, the duration of symptoms, joint misalignment, overuse due to work, sports, etc. (which would capture patient-reported data regarding OA flare ups); (iii) recommendations for enrolling patients with at least >40 mm on the VAS scale (0–100 mm); (iv) using the absolute change in validated pain scales as the primary endpoint for the evaluation of symptom-modifying drugs; and (v) using joint-space narrowing measured by standardized radiographic measures as an indications for structure-altering drugs even though the correlation between structural changes and patient outcomes is weak. Indeed, the FDA issued draft guidance on the basis of new data submitted by invited OA experts that showed a correlation of structural endpoint changes with patient symptoms and pain [233]. There is recognition by regulators that radiographic changes or those identified by MRI may not translate into clinically meaningful outcomes for patients, and there is an openness to consider other biomarkers that better correlate structural changes with patient outcomes [234]. Thus, clinical trials should incorporate traditional pain, function, and structural endpoints using validated patient-reported outcome instruments and radiological and imaging modalities and include a large number of secondary endpoints that can help capture local and systemic inflammatory changes and changes to cartilage degradation biomarkers, as we did [119]. Additionally, detailed collection and reporting of basal and follow-up patient characterization to include clinical phenotype and molecular endotype (using PROMs, biomarkers, and imaging and biomechanical measurements) will be critically important in parsing out the effects of MSC treatment with respect to different patient clinical phenotypes and disease endotypes in subsequent post hoc and meta-analyses.

Cost is another consideration for the commercialization of successful MSC therapeutic products. The extent of the effect size and whether there are symptom-modifying or disease-modifying effects (as evaluated by a combination of PROMs, imaging, and biomarker and biomechanic assays) will determine the willingness to pay of the health care system and market. Using patient survey-based methods, Piuzzi et al. obtained pricing information for same-day minimally manipulated autologous cell preparations from 65 centers in the USA and found that the mean price was US$ 5156 ± 2446 for a single treatment [235]. Many of these procedures are not approved by regulatory bodies and have no evidence or mixed levels of evidence of efficacy, are usually marketed directly to consumers, and require out-of-pocket pay. Even with these caveats, these findings showcase a willingness among OA patients to pay for these therapies. Specifically, a S. Korea MSC(CB) product for treatment of degenerative arthritis showed that the incremental cost-effectiveness ratio (ICER) in terms of quality-adjusted life-years (QALYs) was US$ 16,812 per QALY when compared to microfracture [182], a surgical procedure involving drilling holes in the bone and enabling clot formation and endogenous cartilage repair. Efficacy data were obtained from 5-year follow-up from RCTs, while health care utilization costs were based on the S. Korean health care system. Even with these caveats, the ICER is lower than cost-effectiveness thresholds in different countries [236], typically benchmarked to the gross domestic product (GDP), including the USA (US$ 50 000), Canada (US$ 15–22,000) and UK (US$ 24–36,000). This lends optimism to having a reimbursable MSC product for OA, particularly if longer-term effects can be demonstrated. Thus, the follow-up of patients (beyond the typical 12-month endpoints) and understanding changes in disease trajectory, including further requirements for surgical interventions (and the time scale of those), will be important data for sponsors to obtain to support health cost economic analyses. Ultimately, a successful MSC therapeutic product is one that is not only authorized by regulators but also routinely used by clinicians and is reimbursed by health care payers.

## Summary

Even with 15 completed RCTs, the sample size of 610 treated patients (with MSCs and control treatments) is relatively small to reach definitive conclusions on optimal dosing, baseline patient endotype and phenotype, delivery strategies, MSC sourcing, manufacturing, and overall treatment efficacy. Nonetheless, the findings of meta-analyses and our own analyses revealed positive effects on pain, function and symptoms at final follow-up endpoints in MSC-treated patients relative to baseline (12/15). In 11/15 RCTs, there were improvements in these metrics relative to the control group (HA, PRP, or surgical intervention) at 6 months or longer, depending on the dose of MSC, MSC basal fitness, the baseline clinical phenotype of the patient and the choice of the control comparator. MSCs have also demonstrated chondroprotective or regenerative effects on cartilage in 18/21 clinical studies, including 11/15 RCTs. MSCs have multimodal mechanisms of action, including chondroprotective effects, effects on cartilage repair and ECM anabolism, and pro-angiogenic, antifibrotic, anti-inflammatory and immunomodulatory effects, but there is a dearth of clinical data to confirm these mechanisms in OA. Defining baseline patient disease endotype along with clinical phenotyping will help identify and stratify patients who are receptive to receiving MSC therapies. Patient endotyping will require combinatorial tools, including different types of omics data, levels of systemic and local inflammatory markers, and immune cell subpopulations, which will need to be correlated with patient clinical outcomes and structural changes to validate them as sensitive and reliable biomarkers of patient endotypes. The episodic nature of OA further confounds the categorization of patients by clinical phenotype and/or disease endotyping but should be factored in to better understand the highly heterogeneous, personalized and temporally variable nature of disease progression. The selection of basally fit MSC products involves selecting MSCs from a host of donors that display CQAs that are relevant in the context of OA, including immunomodulatory properties, angiogenic properties and tissue remodeling properties. CQAs are modifiable by culture expansion and cryopreservation, and hence, care needs to be taken to optimize these parameters to maintain the CQAs of MSC products for clinical use. Ultimately, basally fit MSC products may be genetically engineered or nongenetically primed to be “fit-for-OA" with enhanced immunomodulatory and/or pro-angiogenic and/or cartilage-reparative properties to override reliance on donor heterogeneity and culture parameters. A combination of basally fit MSCs that are applied to a subset of knee OA patients who are receptive to receiving MSC treatments (based on disease endotyping and clinical phenotyping) will result in successful outcomes. Statistical modeling will need to be used to collect data from different trials to show the correlation between patient basal status, MSC basal fitness status and ultimately responder/nonresponder status. To enable this, larger sample sizes (for example, a 1020 patient Phase III trial to investigate the safety and efficacy of INVOSSA™, a gene-engineered cell therapy product for OA [237]) or, alternatively, a pooled, registry-based approach with agreements in place to facilitate open sharing of collected data will be necessary to overcome the significant challenges posed by this multifactorial disease, which has proven refractory to multiple therapeutics. Clinical trials will need to be designed with this in mind and actively strive to collect and report on multiple variables to enable patient endotyping/clinical phenotyping and follow-up involving patient outcomes, structural changes (imaging) and biomarkers. Longer periods of follow-up will increase confidence in the effects and the duration of the effects on outcome measures and enable cost justification of MSC therapies. These approaches will entail higher costs for clinical trial sponsors and may behoove a collaborative, consortium-based approach that the FDA has signaled a willingness to entertain [238]. Ultimately, larger, well-designed, targeted clinical trials with extensive characterization of the OA patient population and the MSC product or pooled data from several clinical trials through nontraditional consortium approaches will be needed to advance MSC therapies in OA.
